# Determining the impact of the COVID-19 pandemic on the consumption of antibiotics in Shaanxi province, China: an interrupted time-series analysis

**DOI:** 10.3389/fpubh.2025.1475207

**Published:** 2025-02-19

**Authors:** Wenjing Ji, Yifei Zhao, Jiaxi Du, Hang Zhao, David J. McIver, Dan Ye, Kangkang Yan, Xiaolin Wei, Yu Fang

**Affiliations:** ^1^Department of Pharmacy Administration and Clinical Pharmacy, School of Pharmacy, Xi'an Jiaotong University, Xi'an, China; ^2^Institute for Global Health Sciences, University of California, San Francisco, San Francisco, CA, United States; ^3^Department of Pharmacy, Xi'an No.3 Hospital, The Affiliated Hospital of Northwest University, Xi'an, China; ^4^Dalla Lana School of Public Health, University of Toronto, Toronto, ON, Canada

**Keywords:** antibiotics, consumption, expenditure, COVID-19, interrupted time series, China

## Abstract

**Background:**

The COVID-19 pandemic, as well as associated prevention and control measures, have impacted the entire healthcare system, including the use patterns of medicine. However, the impact of COVID-19 on the use of antibiotics has not yet been extensively evaluated in China. This study aimed to evaluate the impact of the pandemic on the consumption and expenditure of antibiotics in public healthcare institutions in Shaanxi Province, China.

**Methods:**

We used longitudinal data from the Shaanxi provincial drug procurement database in this study. An interrupted time-series analysis was performed to evaluate the effects of COVID-19 on consumption and expenditures of antibiotics in public healthcare institutions in Shaanxi from 1 January 2017 to 31 December 2020. Antibiotic consumption was expressed as the number of defined daily doses (DDDs) per 1,000 residents per day (DIDs), based on the population of Shaanxi Province at the end of each year from the National Bureau of Statistics. The pre-pandemic period was from January 2017 to January 2020, and the post-pandemic period was from February 2020 to December 2020.

**Results:**

A declining trend in antibiotic consumption was observed immediately following the COVID-19 pandemic (*β*2 = −4.099; *p* < 0.001). Regarding the route of administration, a larger decrease in the consumption of oral antibiotics was observed compared to parenteral (*β*2 = −3.056; *p* < 0.001). The decrease in antibiotic consumption in the Watch category (*β*2 = −2.164; *p* < 0.001) was larger than in the Access category (*β*2 = −1.773; *p* < 0.001), and penicillins (J01C) (*β*2 = −1.261; *p* < 0.001) showed a higher decline than cephalosporins (J01D) (*β*2 = −1.147; *p* < 0.001). An increasing trend of broad-spectrum antibiotic consumption was observed after the onset of the pandemic (*β*3 = 0.021; *p* = 0.015). Expenditures on and consumption of antibiotics follow essentially the same trend.

**Conclusion:**

Despite an initial decline in antibiotic consumption at the start of the COVID-19 pandemic, it had returned to prior levels by the end of 2020. Findings underscore the continued importance of antibiotic stewardship initiatives.

## 1 Introduction

The increasing trend of antimicrobial resistance (AMR) seriously threatens global public health, economic growth, and economic stability. Inappropriate use of antibiotics, including misuse and overuse, is the key driver of this resistance (1, 2). It was estimated that in the absence of effective strategies to control antimicrobial resistance, more than 10 million people worldwide will die each year from drug-resistant infections by 2050 (3). Due to the slow development of new alternative antibiotics, the prudent use of existing antibiotics remains essential to prevent and control the surge in bacterial resistance (4).

To assist antibiotic stewardship programs with global metrics and indicators for appropriate use, the WHO published an Access, Watch, and Reserve antibiotics (AWaRe) classification of antibiotics (5), which recommends Access antibiotics represent at least 60% of total antibiotic consumption. In the AWaRe classification, access antibiotics have a narrow spectrum of activity and a good safety profile in terms of side effects. Watch antibiotics are broad-spectrum antibiotics and are recommended as first-choice options for patients with more severe clinical presentations or for infections where the causative pathogens are more likely to be resistant to Access antibiotics. Reserve antibiotics are the last-choice antibiotics used to treat multidrug-resistant infections. The reported rate of antibiotic consumption and AMR was very high in China between 2000 and 2015 (6). Moreover, the total antibiotic usage in China’s hospitals increased by 39.6% during the same period; in particular, Access antibiotics accounted for 19.4%, which is well below the recommended target of WHO of 60% at the national level (7).

To promote the rational use of antibiotics, the National Health Commission of China issued a series of broad regulations on antibiotic use between 2011 and 2016, including the establishment of a technical support framework, management and surveillance systems (8), and setting antibiotic use as performance evaluation indicators for tertiary public hospitals (9). The most important aspect of antimicrobial management is antimicrobial classification management, in which antibacterial agents are divided into non-restricted, restricted, and special categories based on clinical efficacy, drug safety, impact on bacterial resistance, and other factors, including price (10). This classification refers to the WHO AWaRe list, and it has been adjusted according to the use of antibiotics and bacterial resistance in China. Although an overall reduction in antibiotic use following these efforts was observed (11–13), the China Antimicrobial Surveillance Network (CHINET) reported that more than 50% of *Escherichia coli* strains were resistant to third-generation cephalosporin antibiotics in 2023, and 29.6% of *Staphylococcus aureus* strains were resistant to methicillin (14). High rates of resistance to the antibiotic meropenem were also observed for *Acinetobacter baumannii* (73.7%), *Klebsiella pneumoniae* (26%), and *Pseudomonas aeruginosa* (17.4%) (15). The incidence of carbapenem-resistant Gram-negative bacteria has also risen, leading to potentially fatal infections, particularly when these bacteria demonstrate resistance to last-resort antibiotics (15, 16).

On the other hand, the WHO declared coronavirus disease 2019 (COVID-19) a pandemic on 11 March 2020. By then, COVID-19 had spread all over the world and impacted healthcare delivery, access to healthcare, infectious diseases managed with antibiotics, and vaccination (17, 18). A recent meta-analysis reported that COVID-19 significantly increased antibiotic resistance, particularly Gram-negative bacteria, in hospital settings (19). Macera et al. (20) found an increased consumption of broad-spectrum antibiotics due to the interruption of the antimicrobial stewardship program during COVID-19 in Southern Italy. Similar findings have been reported from India (21) and Spain (22). In contrast, community-based studies conducted in Portugal (23), Wales (24), Canada (25), Spain (26), the UK (27), China (28, 29), and most European countries (30) reported significant decreases in consumption of total antibiotics. A study conducted in a tertiary care center in Lebanon showed that total carbapenem consumption decreased by 71.2% in 2020 compared with 2015–2019 (21).

The patterns of antibiotic use may have changed considerably during the COVID-19 pandemic globally (28, 29), which needs further investigation. Meanwhile, there is inadequate evidence regarding the overall utilization and the impact of the COVID-19 pandemic on antibiotic consumption in China, particularly in Northwest China, considering that the use of antibiotics and the effects of the pandemic may vary by province and region due to socioeconomic conditions, distribution of medical resources, and antibiotic resistance level. We therefore retrospectively analyzed antibiotic consumption in Shaanxi Province and quantified the trends of antibiotic use before and after the COVID outbreak to evaluate the impact of the pandemic. The findings of this study will fill the gap in the literature about antibiotic use in Northwest China and add evidence for the global assessment regarding the impact of COVID-19 on antibiotic use.

## 2 Materials and methods

### 2.1 Study setting and design

We used an interrupted time-series design on a provincial database of antibiotic procurement records from 1 January 2017 to 31 December 2020. We examined the effects of the COVID-19 pandemic by comparing pre- and post-pandemic trends in antibiotic consumption and hospital expenditures on antibiotics. Differences in consumption and expenditure patterns between WHO AWaRe antibiotic classifications were also examined.

### 2.2 Data source and collection

Data for this study were extracted from the centralized drug bidding procurement and supply chain system of Shaanxi. The Chinese government established the centralized drug bidding procurement and supply chain system at the provincial level across China in 2009 to improve the rational use of antibiotics, control increasing expenditure on medicines, support the essential medicine policy, and regulate medicine procurement behaviors. As of 2015, all public healthcare institutions in China are required to purchase medicines through the provincial centralized drug procurement platform (31). The system provides services for healthcare facilities to order, store, and deliver drugs.

Procurement data on antibiotics were collected every month, including information on the following variables: the type of healthcare institution, procurement date, generic name, dosage form, specification, pack size, manufacturer, price per unit, purchasing unit, purchase volume, and purchase expenditures.

### 2.3 Intervention

We defined our interventions as the onset of the COVID-19 pandemic in China in January 2020. The outbreak of COVID-19 was first reported in December 2019 in Wuhan, Hubei Province, China. Following a rapid spread across the country, the first Shaanxi case is confirmed in January 2020 (32).

### 2.4 Outcomes measures

To describe the use of antibiotics in this study, antibiotic procurement was calculated and expressed as the number of defined daily doses (DDDs) per 1,000 residents per day (DIDs) based on the population of Shaanxi Province at the end of each year from the National Bureau of Statistics (33). It was calculated as the total DDDs divided by the population total, multiplied by 1,000. DDDs are the total consumption of antibiotic agents divided by DDDs, which was calculated as the number of unit strengths (g) × pack size×amount/DDD (g/IU). DDDs are the average daily maintenance dose of medication for adults for primary therapeutic purposes and was developed by the WHO Collaborating Centre for Drug Statistics Methodology. The value of DDD can reflect drug dynamics and drug use patterns over months; higher values indicate greater selection tendency and dosage. Antibiotic expenditure was assessed using the total monthly expenditure for individual antibiotics (US dollars). The expenditure was calculated by adding up the cost for each procurement, which was equal to the quantity purchased per order multiplied by the unit price.

Antibiotic use data were categorized by the Anatomical Therapeutic Chemical (ATC) classification J01 (34). The included quality indicators (QIs) mainly reference the European Surveillance of Antimicrobial Consumption [ESAC, recognized as an internationally comparable methodology (35)] and the AWaRe classification, developed by the WHO Expert Committee on the Selection and Use of Essential Medicines based on potential antimicrobial resistance, economy, and other factors (36), for measuring antibiotic procurement in order to better reflect the consumption of antibiotic agents in this study (Table 1). We included 10 ESAC QIs (37), whereas the remaining 2 QIs involving seasonal antibiotic use were not included because they are of little relevance to the objective of this study to assess the impact of pandemics on antibiotic procurement. In addition, we analyzed antibiotics according to inclusion in the classification of essential drugs, restricted and different areas. Supplementary Table S1 provides a description of these indicators of antibiotic consumption, and results are provided in Supplementary Figures S1–S5. Supplementary Table S2 provides the classification of antibiotics used in this study. Supplementary Figures S7–S9 show the results of the expenditure on main-classification antibiotics.

**Table 1:** Description of quality indicators of antibiotic consumption.

No.	Indicator	Description
1	Access	Consumption of Access group antibiotics according to the 2019 AWaRe categorization
2	Watch	Consumption of Watch group antibiotics according to the 2019 AWaRe categorization
3	Reserve	Consumption of Reserve group antibiotics according to the 2019 AWaRe categorization
4	Oral	Consumption of oral antibiotics
5	Parenteral	Consumption of parenteral antibiotics
6	J01	Consumption of antibiotics for systemic use (J01)
7	J01C	Consumption of penicillins (J01C)
8	J01D	Consumption of cephalosporins (J01D)
9	J01F	Consumption of macrolides, lincosamides, and streptogramins (J01F)
10	J01M	Consumption of quinolones (J01M)
11	J01CE	Percentage of *β*-lactamase-sensitive penicillins (J01CE) in total use of J01
12	J01CR	Percentage of combination of penicillins, including *β*-lactamase inhibitor (J01CR) in total use of J01
13	J01(DD + DE)	Percentage of combination of the third and fourth generations of cephalosporins [J01(DD + DE)] in total use of J01
14	J01MA	Percentage of fluoroquinolones (J01MA) in total use of J01
15	J01B/N	Ratio of the consumption of broad {J01[CR + DC + DD+(F-FA01)]} to the consumption of narrow-spectrum penicillins, cephalosporins, and macrolides [J01(CE + DB + FA01)]

^*^Indicators 1–10 are the use of relevant antibiotics, expressed as differences in differences (DIDs), and indicators 11–15 are the relative contributions expressed as a percentage.

### 2.5 Statistical analysis

An interrupted time-series analysis (ITS), a robust quasi-experimental design, was used to evaluate the impact of the COVID-19 pandemic on the utilization of antibiotics. An ITS assesses the effect of the intervention by constructing an interrupted linear regression model, including the change in the level before and after the intervention point in time and whether the slope of the decline or increase in procurement over time changed after the intervention was implemented and thus assesses the effect of the intervention on the outcome variable (38). Monthly antibiotic procurement and expenditure were the units of analysis, with January 2017 to December 2019 (36 months) defined as the pre-pandemic period and February 2020 to December 2020 (11 months) as the following period of the beginning of the pandemic. We set the lag to 1 month (January 2020) in the interrupted time-series analysis.

The segmented regression models were used to analyze pre- to post-pandemic changes in the use of antibiotics overall, by monthly use, and by antibiotic classification. We also analyzed changes in antibiotic procurement by quality indicators as listed in Table 1. The Durbin–Watson statistic (39) was used to test whether there was autocorrelation, and the Dicky–Fuller statistic was used to examine seasonal fluctuations. The time–trend scatter plot revealed seasonal fluctuations in the data, with winter usage significantly higher than that of other seasons. Based on our data structure and previous research (40), we set a dummy variable “season” to control for the extreme values of antibiotic use. The seasonal variable was set to 1 for January, November, and December and set to 0 for other months. The regression model for each outcome is as follows:

$$\begin{array}{l} Y_{t}=\beta_{0}+\beta_{1}\times {\mathrm{time}}_{t}+\beta_{2}\times {\mathrm{intervention}}_{t}+\beta_{3}\\ \;\times \mathrm{time}\;{\mathrm{after intervention}}_{t}+\beta_{4}\times \mathrm{season}+et \end{array}$$

Where:

Y_t_ is the outcome in time t; time_t_ is a continuous variable indicating time in months at time t from the start of the observation period; intervention_t_ is an indicator for time t occurring before (intervention = 0) or after (intervention = 1); time after intervention_t_ is a continuous variable counting the number of months after the intervention at time t; coded 0 before the intervention and (t-37) after the intervention; season is a seasonal variable, set to 1 for November, December, and January, and set to 0 for other months; *β*_0_ estimates the baseline level of the outcome; *β*_1_ estimates the baseline trend; *β*_2_ estimates the level change in the outcome after the intervention; *β*_3_ estimates the trend change in the outcome after the intervention; *β*_4_ estimates the potential seasonality effect; et represents the random variability not explained by the model at time t.

All statistical tests were two-sided, and we considered an outcome significant when the *p* < 0.05. All statistical analyses were performed in RStudio version 4.3.1. Supplementary Table S3 provides full regression data of antibiotic procurement analyzed in this study. Supplementary Table S4 shows full regression data of antibiotic expenditures analyzed in this study.

## 3 Results

The total number of public healthcare institutions that reported data in the centralized drug bidding procurement and supply chain system was 2,292 across all the cities of Shaanxi Province between 1 January 2017 and 31 December 2020, with 256 urban institutions, 290 county-level institutions, and 1,746 primary healthcare institutions.

### 3.1 Usage patterns of systemic antibiotics via different administration routes

A sharp and immediate decline in overall monthly antibiotic consumption was observed after the outbreak of the COVID-19 pandemic (*β*_2_ = −4.099; *p* < 0.001) (Table 2), according to the regression equation:

$$\begin{array}{l} Y_{t}=5.394+0.103\times {\mathrm{time}}_{t}-4.099\times {\mathrm{intervention}}_{t}+0.124\\ \;\times \mathrm{time}\;{\mathrm{after intervention}}_{t}+1.473\times \mathrm{season}+et \end{array}$$

**Table 2:** Interrupted time-series analysis of antibiotic consumption with the onset of the COVID-19 pandemic as an intervention.

	Change in level (*β*_2_)			Change in level (*β*_3_)			Autocorrelation coefficient
	Value	*p*-value	95% CI	Value	*p*-value	95% CI	
Use of relevant antibiotics (expressed in DIDs)							
J01	−4.099	0.000	(−6.153, −2.045)	0.124	0.315	(−0.122, 0.370)	1.870
J01C	−1.261	0.000	(−1.790, −0.732)	0.066	0.063	(−0.004, 0.135)	2.012
J01D	−1.147	0.000	(−1.759, −0.534)	0.042	0.204	(−0.024, 0.109)	1.544
J01F	−0.869	0.000	(−1.286, −0.451)	−0.005	0.868	(−0.059, 0.050)	1.715
J01M	−0.320	0.024	(−0.596, −0.045)	0.009	0.634	(−0.029, 0.047)	2.161
Access	−1.773	0.000	(−2.591, −0.956)	0.084	0.109	(−0.019, 0.188)	1.957
Watch	−2.164	0.000	(−3.299, −1.030)	0.032	0.627	(−0.099, 0.162)	1.743
Reserve	0.003	0.503	(−0.005, 0.010)	0.000	0.547	(−0.001, 0.001)	1.837
Oral	−3.056	0.000	(−4.506, −1.606)	0.114	0.189	(−0.058, 0.286)	1.926
Parenteral	−1.043	0.002	(−1.671, −0.416)	0.010	0.799	(−0.068, 0.088)	1.636
Relative contributions							
J01CE	−0.004	0.006	(−0.007, −0.001)	0.001	0.031	(0.000, 0.001)	2.418
J01CR	−0.004	0.431	(−0.015, 0.007)	0.002	0.013	(0.000, 0.003)	1.545
J01(DD + DE)	0.005	0.508	(0.011, 0.021)	−0.001	0.436	(−0.003, 0.001)	1.388
J01MA	0.028	0.000	(0.016, 0.040)	−0.002	0.017	(−0.003, 0.000)	2.167
J01B/N	0.041	0.587	(−0.110, 0.191)	0.021	0.015	(0.004, 0.038)	2.083

**Figure 1:**
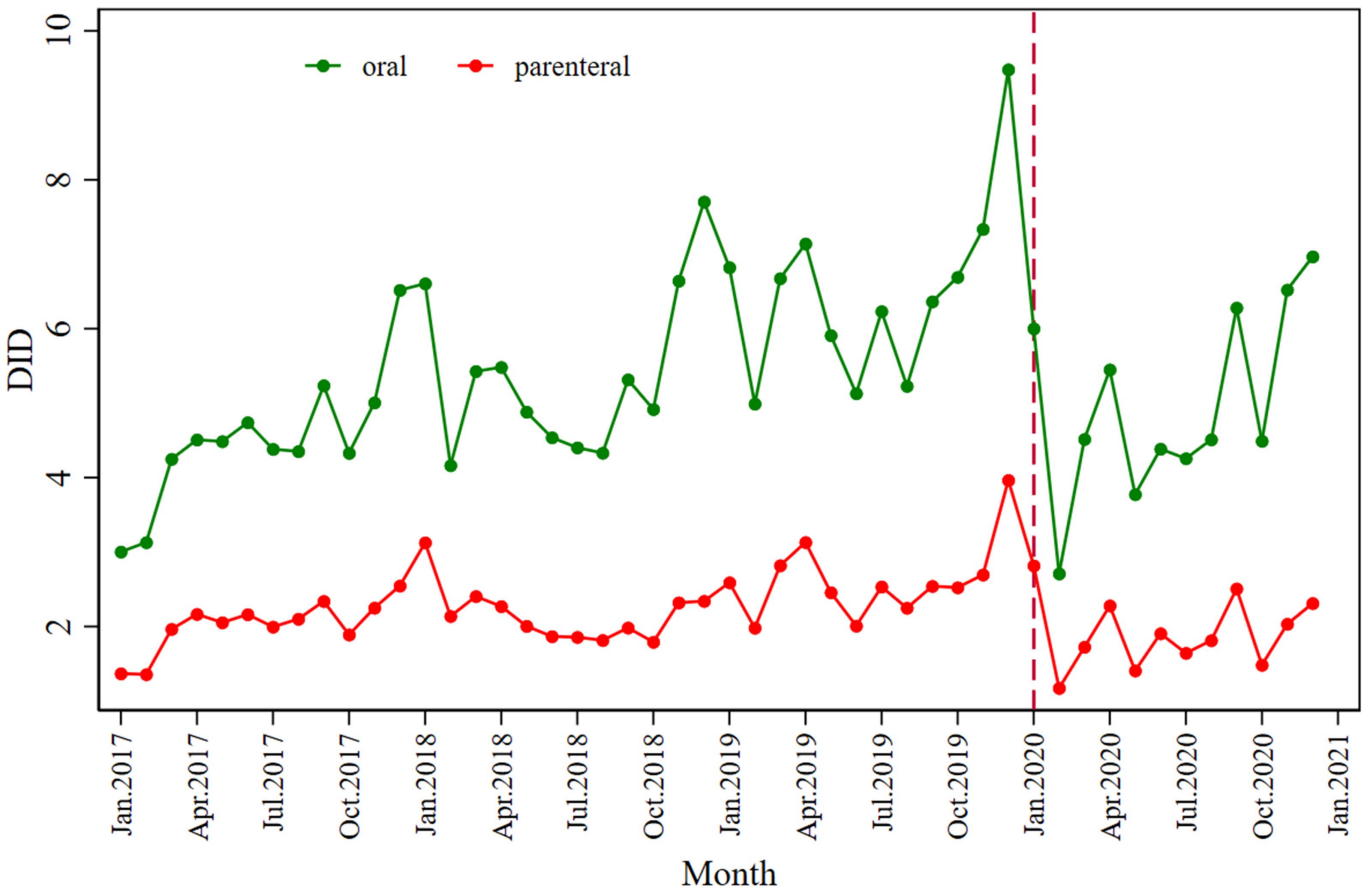
Monthly antibiotic consumption of oral and parenteral antibiotics.

The absolute value of decline is −3.775. However, antibiotic consumption showed a slightly increasing trend after COVID-19 began, from the intervention in January 2020 to December 2020. Regarding the route of administration, both the consumption of oral (*β*_2_ = −3.056; *p* < 0.001) and parenteral antibiotics (*β*_2_ = −1.043; *p* = 0.002) decreased immediately following the intervention; the values of the declines were −2.790 and −0.016. The details regarding antibiotic consumption can be seen in Figure 1 and Table 2.

**Figure 2:**
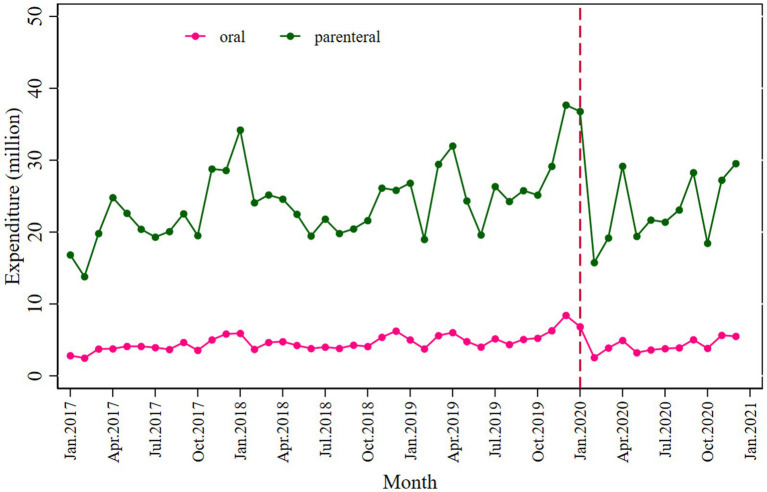
Monthly antibiotic expenditure for oral and parenteral antibiotics.

Similarly, a significant decrease in total antibiotic expenditure was observed after the COVID-19 pandemic outbreak (*β*_2_’ = −9.134; *p* = 0.030), with a larger decline in parenteral antibiotic expenditure (*β*_2_’ = −7.027; *p* = 0.050) than oral antibiotics (*β*_2_’ = −2.122; *p* = 0.002), as shown in Figure 2 and Table 3.

**Table 3:** Interrupted time-series analysis of antibiotic expenditures with the onset of the COVID-19 pandemic as an intervention.

	Change in level (*β*_2_’)			Change in level (*β*_3_’)			Autocorrelation coefficient
	Value	*p*-value	95% CI	Value	*p*-value	95% CI	
Use of relevant antibiotics (expressed in DIDs)							
J01	−9.134	0.030	(−17.345, −0.933)	0.161	0.738	(−0.801, 1.122)	1.516
J01C	−1.803	0.015	(3.234, −0.372)	0.039	0.638	(−0.127, 0.205)	1.547
J01D	−5.558	0.043	(−10.927, −0.189)	0.218	0.501	(−0.430, 0.867)	1.549
J01F	−0.694	0.003	(−1.140, −0.249)	−0.039	0.182	(−0.098, 0.019)	1.232
J01M	−0.241	0.407	(−0.821, 0.339)	−0.045	0.258	(−0.124, 0.034)	1.744
Access	−1.655	0.005	(−2.796, −0.514)	0.132	0.066	(−0.009, 0.274)	1.570
Watch	−6.834	0.040	(−13.347, −0.320)	0.012	0.976	(−0.759, 0.782)	1.519
Reserve	0.021	0.759	(−0.115, 0.157)	0.006	0.561	(−0.016, 0.028)	2.130
Oral	−2.122	0.002	(−3.389, −0.836)	0.040	0.573	(−0.103, 0.183)	1.670
Parenteral	−7.027	0.050	(−14.048, −0.005)	0.120	0.773	(−0.713, 0.954)	1.515
Relative contributions							
J01CE	0.000	0.371	(0.000, 0.000)	0.000	0.000	(0.000, 0.000)	1.537
J01CR	−0.004	0.194	(−0.011, 0.002)	0.001	0.009	(0.000, 0.000)	1.323
J01(DD + DE)	0.004	0.790	(−0.024, 0.032)	−0.002	0.181	(−0.006, 0.001)	1.965
J01MA	0.024	0.002	(0.009, 0.039)	−0.003	0.003	(−0.004, −0.001)	1.309
J01B/N	1.419	0.270	(−1.141, 3.979)	−0.068	0.671	(−0.388, 0.252)	1.431

### 3.2 Antibiotic usage based on the AWaRe classification

**Figure 3:**
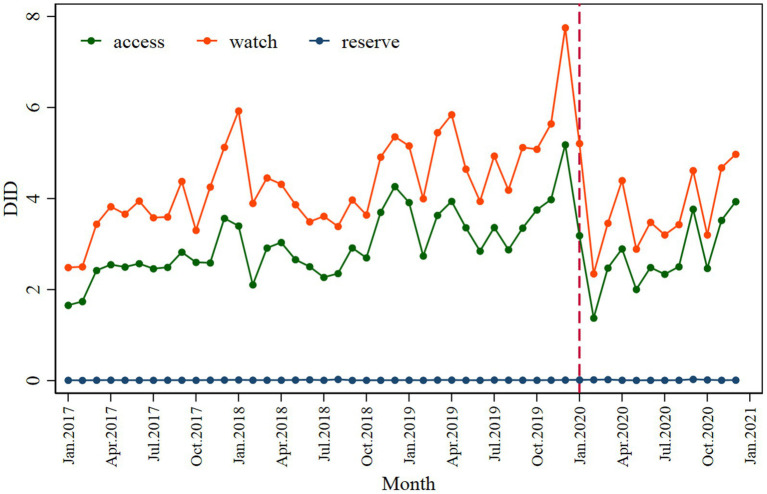
Monthly antibiotic consumption by access, watch, and reserve antibiotics.

A significant decrease in the Access (*β*_2_ = −1.773; *p* < 0.001) and Watch (*β*_2_ = −2.164; *p* < 0.001) categories was observed following the intervention, with a slightly inclined trend observed in the post-COVID-19 period (Figure 3 and Table 2). The values of the declines were −1.608 and −1.019.

**Figure 4:**
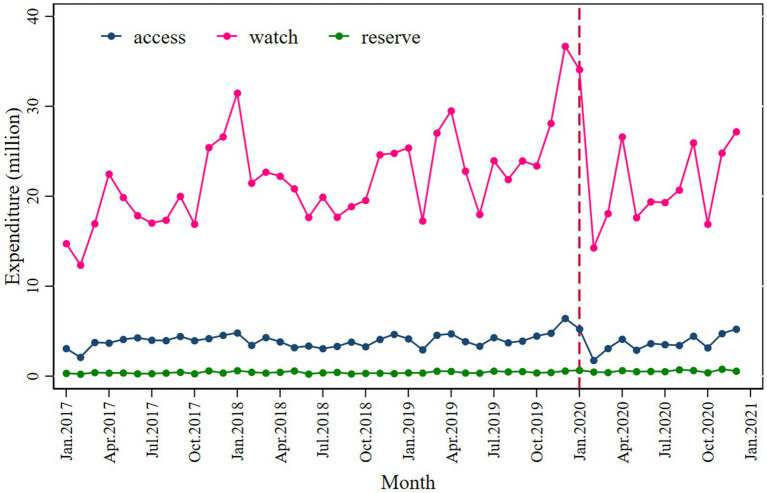
Monthly antibiotic expenditure for access, watch, and reserve antibiotics.

Expenditure on antibiotics decreased significantly in the Watch category (*β*_2_’ = −6.834; *p* = 0.040) compared to the Access (*β*_2_’ = −1.655; *p* = 0.005) following the intervention (Figure 4 and Table 3).

### 3.3 Utilization of antibiotics based on the ATC classification

**Figure 5:**
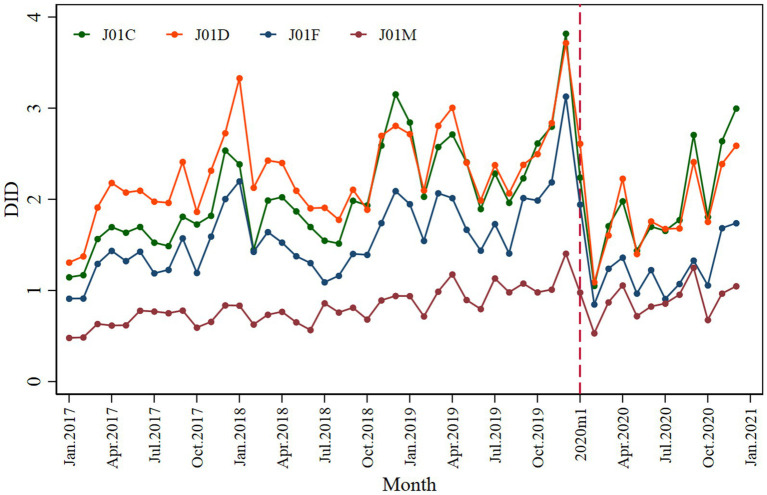
Monthly antibiotic consumption of penicillins (J01C), cephalosporins (J01D), macrolides, lincosamides, streptogramins (J01F), and quinolone (J01M).

Penicillins (J01C), cephalosporins (J01D), macrolides, lincosamides, and streptogramins (J01F) showed a significantly declined trend when the pandemic COVID-19 occurred; however, during the post-pandemic intervention period, penicillins and cephalosporins showed an inclined trend, as shown in Figure 5. The penicillins (J01C) (*β*_2_ = −1.261; *p* < 0.001) showed a greater initial decline than cephalosporins (J01D) (*β*_2_ = −1.147; *p* < 0.001), macrolides, lincosamides, streptogramins (J01F) (*β*_2_ = −0.869; *p* < 0.001), and quinolones (J01M) (*β*_2_ = −0.320; *p* = 0.024) (Table 2).

Cephalosporins (J01D) showed a larger decline (*β*_2_’ = −5.558; *p* = 0.043) in terms of expenditure than penicillins (J01C) (*β*_2_’ = −1.803; *p* = 0.015), macrolides, lincosamides, and streptogramins (J01F) (*β*_2_’ = −0.694; *p* = 0.003) (Supplementary Figure S7 and Table 3).

### 3.4 Monthly relative contributions of different antibiotics to systemic antibiotic usage

**Figure 6:**
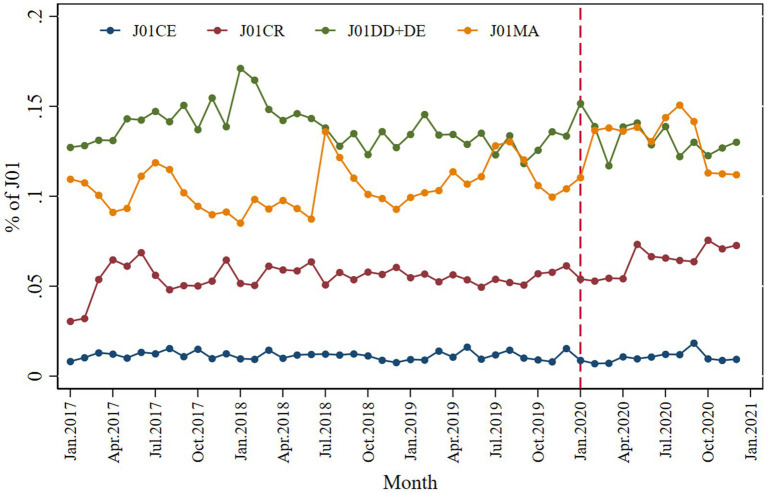
Monthly relative consumption contributions of J01CE, J01CR, J01DD + DE, and J01MA to total use of systemic antibiotics.

This study analyzed the monthly contributions of the following four antibiotics to systemic antibiotics: J01CE (ß-lactamase-sensitive penicillins), J01CR (combinations of penicillins with ß-lactamase inhibitors), J01 (DD + DE) (third- and fourth-generation cephalosporins), J01MA (fluoroquinolones). Regarding the relative contribution to systematic antibiotic usage in the entire data set, third- and fourth-generation cephalosporins accounted for 14% of total antibiotic usage, followed by fluoroquinolones (12%) and a combination of penicillins with ß-lactamase inhibitors (5%), as shown in Figure 6. A slight decrease in the relative contribution of ß-lactamase-sensitive penicillins was observed immediately after the pandemic of COVID-19 (*β*_2_ = −0.004; *p* = 0.006) compared to the systematic use of antibiotics. While fluoroquinolones positively contributed to systematic usage (*β*_2_ = 0.028; *p* < 0.001), the contribution was small (Table 2).

A negligibly small effect of COVID-19 was observed on antibiotics in terms of relative contribution to expenditure (Supplementary Figure S8 and Table 3).

### 3.5 Impact on the ratio of consumption of broad- and narrow-spectrum antibacterials

**Figure 7:**
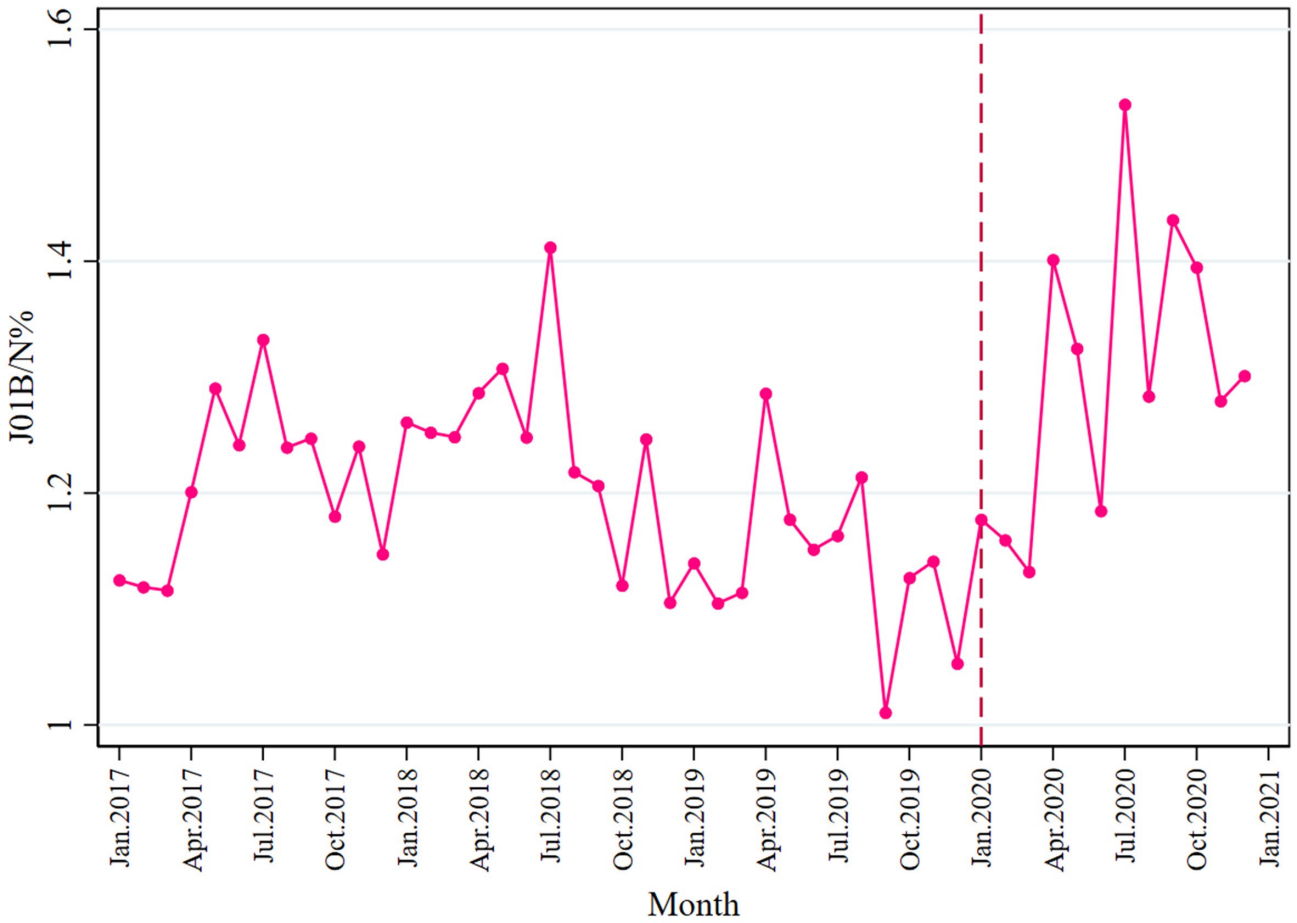
Ratio of consumption of broad- and narrow-spectrum antibiotics.

Overall, the consumption of broad-spectrum antibiotics was higher than the narrow spectrum, as most ratio values of J01B/N were above 1.0 (Figure 7). In the post-pandemic period, a slight increase was seen in broad-spectrum antibiotic consumption (*β*_3_ = 0.021; *p* = 0.015). Detailed information is provided in Table 2. No change was observed before and after the pandemic in terms of expenditure, as shown in Table 3 and Supplementary Figure S9.

### 3.6 Consumption of antibiotic based on other classifications

Regardless of whether antibiotics were classified as essential, consumption of both essential and non-essential antibiotics declined, though essential antibiotics (*β*_2_ = −2.856; *p* < 0.001) declined more than non-essential (*β*_2_ = −1.235; *p* = 0.011) antibiotics (Supplementary Table S3 and Supplementary Figure S1). Restricted antibiotics (*β*_2_ = −0.432; *p* = 0.024) declined more than unrestricted antibiotics (*β*_2_ = −3.670; *p* < 0.001) (Supplementary Table S3 and Supplementary Figure S3). Special antibiotics are not significant. Primary antibiotics (*β*_2_ = −2.734; *p* < 0.001) also declined more than urban antibiotics (*β*_2_ = −1.249; *p* = 0.008) (Supplementary Table S3 and Supplementary Figure S5), and county antibiotics is not significant.

In contrast to changes in consumption, expenditure on urban antibiotics (*β*_2_’ = −5.853; *p* = 0.023) declined more than primary antibiotics (*β*_2_’ = −1.399; *p* = 0.003) (Supplementary Table S4 and Supplementary Figure S6).

## 4 Discussion

We found a sharp decrease in the consumption of and expenditure on antibiotics at the start of the COVID-19 pandemic in Shaanxi, which gradually recovered to near pre-COVID-19 levels by December 2020. An abrupt decline was observed in the consumption of penicillin and cephalosporins when the pandemic started, but consumption then increased in the later part of the pandemic. By antibiotic category, a greater increase in the consumption of the Watch category of antibiotics was observed than in the Access category during the same time, and both declined by the end of the dataset. The consumption of broad-spectrum antibiotics increased more during the pandemic than narrow-spectrum antibiotics. This longitudinal observational study addresses a research gap in the impact of COVID-19 on antibiotic consumption trends in Shaanxi, China, by providing a comprehensive analysis.

In the current study, an initial sharp decline in antibiotic consumption was observed with the onset of the COVID-19 pandemic, which might be due to non-pharmaceutical interventions for preventing and controlling COVID-19, including lockdown overlapping Chinese Spring Festival and restrictions on traveling and gatherings. The National Health Commission of China issued technical guidelines for the prevention and control of novel coronavirus infection in healthcare institutions (First Edition) on 23 January 2020 (41) as well as the first edition of the protocol on prevention and control of novel coronavirus pneumonia based on the latest clinical discoveries and research results (a total of seven editions by the end of 2020) (42). The first case of COVID-19 was confirmed in Shaanxi on 23 January 2020 (32). The provincial government officially issued a notice on strengthening the prevention and control of pneumonia caused by the novel coronavirus on the same day (43). These guidelines encouraged residents to stay at home, extended the Spring Festival holiday and supported online work to reduce personnel turnover, and that healthcare institutions should minimize overcrowding to reduce the risk of hospital infections and the spread of COVID-19.

Meanwhile, due to the panic over the pandemic at the beginning, some people may have decided not to go to the hospital for treatment and obtain antibiotics as usual, unless their illness was severe. On 25 January, the Level I emergency response for public health emergencies was officially launched in Shaanxi; all schools were postponed, and it was recommended that only mild fever and runny nose symptoms should be isolated and observed at home, and disease progression should be monitored (44). All of the above measures may lead to a sharp decline in visits for infections and a reduction in overall antibiotic dispensing in the first months of the pandemic. Several studies have examined the impact of the COVID-19 pandemic on antibiotic consumption in China. Research by Wang et al. (28) found that overall antibiotic consumption in primary healthcare settings (PHSs) decreased by 32.04 and 16.69% in 2020 and 2021, respectively, compared to 2019. Fukushige et al. (45) conducted a systematic review across various countries, including China, reporting a general reduction in community antibiotic consumption in 2020. Wu et al. (46) also found there was a decline in antibiotic utilization rates in hospitals in Southern Sichuan by 2020. These studies are consistent with our observations in this study.

However, we found a gradual increase in antibiotic consumption since March 2020, which was also observed in some other country studies. A national study from the USA evaluated changes in the appropriateness of outpatient antibiotic prescribing after the COVID-19 outbreak; a similar trend was found (47). Moreover, studies in Chile (48) and India (49) found a significant increase in antibiotic use post-pandemic onset (4). The increased antibiotic consumption during the COVID-19 pandemic can be attributed to several factors. Initially, the uncertainty surrounding the management of COVID-19 cases led to a surge in antibiotic sales, especially in low- and middle-income countries, as antibiotics were used for COVID-19 patients despite low rates of bacterial co-infections (50). Furthermore, the impact of the pandemic on antimicrobial use resulted in a substantial rise in intravenous antibiotic consumption, particularly broad-spectrum *β*-lactams and carbapenems (48). These findings underscore the importance of strengthening antimicrobial stewardship efforts and infection prevention and control measures to mitigate the risks associated with increased antibiotic consumption during public health crises such as the COVID-19 pandemic.

This study found that the consumption of the Access and Watch classes of antibiotics declined significantly after the intervention, and we also found that sales of the Watch antibiotics were significantly higher than the Access antibiotics, representing a large gap between the targets recommended by the WHO. The Watch antibiotics carry a higher risk of resistance and are regarded as a second choice, only to be used when the Access antibiotics are ineffective. Future studies should focus on analyzing the appropriateness of the use of Watch antibiotics. We comparatively analyzed a number of studies from other countries and regions and found that the trend of the impact of pandemic outbreaks on antibiotics based on AWaRe classification in these regions is consistent with our study, e.g., Jordan (51), India (49), Spain (52), and Pakistan (53).

The use of broad-spectrum antibiotics was prevalent, and an increase was observed in the consumption of broad-spectrum antibiotics in the post-intervention period. Patients tested as COVID-19 positive in public healthcare institutions were more likely to have broad-spectrum antibiotics during the early pandemic. Our findings are consistent with prior studies in other countries that showed varying trends in the use of broad-spectrum antibiotics during the pandemic. In England, an initial increase trend in broad-spectrum antibiotic prescribing was seen, particularly for respiratory tract infections and otitis media, followed by a gradual decline (54). Another study from Italy reported that during the pandemic, the prescription of broad-spectrum antibiotics was high, especially in respiratory tract infections, although the total antibiotic prescription declined from March 2020 onward (55). The increased use of broad-spectrum antibiotics may be due to diagnostic uncertainty and the prescribing of broad-spectrum antibiotics to cover a range of potential infections during the pandemic. The use of broad-spectrum antibiotics, while beneficial in covering a wide range of pathogens, can lead to increased antibiotic resistance compared to antibiotics that are narrower and more targeted in scope (56, 57). Therefore, limiting the use of broad-spectrum antibiotics in favor of narrow-spectrum options is essential to combat antibiotic resistance and optimize patient outcomes. Despite the fact that antimicrobial stewardship (AMS)—an approach that aims to minimize the use of antibiotics, particularly broad-spectrum antibiotics, while maintaining optimal patient outcomes—is considered imperative to optimize the use of antimicrobial drugs, AMS programs in clinical settings must cope with many challenges. Factors including a reduction in the number of qualified staff available, limits to laboratory supplies and equipment for AMR investigation activities, and a decrease in partnerships and funding for related activities all have the potential to negatively impact AMS programs. To avoid negative consequences for patient outcomes and antimicrobial resistance in the future, particularly during epidemic and pandemic events, emphasis should be given to AMS programs.

To our knowledge, this is the first study assessing antibiotic consumption before and after COVID-19 in public healthcare institutions in Shaanxi Province. Providing information from a provincial procurement database allowed us to have a picture of antibiotic utilization impacted by the pandemic. The current study is subjected to various limitations. First, this study was conducted only in one province; therefore, the findings may not be generalizable to the whole country. Second, we used data up to December 2020 only, and the trend of antibiotic consumption may vary past this date throughout the remainder of the COVID-19 pandemic. Third, the current study only showed the impact of COVID-19 on antibiotic consumption and expenditures, while other factors such as increased burden on the hospital, change in prescribing behavior due to the disease uncertainty, and the prophylactic use of antibiotics to prevent secondary infections may impact antibiotic consumption trends. It is recommended that future studies extend the post-intervention period, incorporate additional mediating variables, and be conducted nationally. Studies assessing the appropriateness of antibiotic prescribing for specific conditions, such as COVID-19 and respiratory infections, are necessary.

## 5 Conclusion

The current study showed that antibiotic consumption abruptly declined when the COVID-19 pandemic started and was rising as of December 2020. The consumption of broad-spectrum antibiotics in Shaanxi Province was high even before the pandemic. Antibiotic stewardship programs are still needed to prevent unnecessary morbidity associated with antibiotic-related adverse events and antimicrobial resistance.

## Data Availability

The original datasets analyzed during the current study are not publicly available due to privacy restrictions but are available from the corresponding author on reasonable request. Requests to access the datasets should be directed to DY, yedan20100910@163.com and KY, 276317800@qq.com.
