# Supplementary material for: Determining the impact of the COVID-19 pandemic on the consumption of antibiotics in Shaanxi province, China: an interrupted time-series analysis
Source: Front Public Health. 2025 Feb 19;13:1475207. doi: 10.3389/fpubh.2025.1475207 (PMC11880026; doi:10.3389/fpubh.2025.1475207)
Supplement: Supplementary file 1 [file Data_Sheet_1.zip › Supplementary _mateial.docx]

Supplementary Material

# Supplementary Materials

## Supplementary Tables

**Table S1**. Description of other indicators of antibiotic consumption.

| **NO** | **Indicator** | **Description** |
| --- | --- | --- |
| 1 | Essential | Consumption of antibiotics included in the National Essential Drug List |
| 2 | Unessential | Consumption of antibiotics not included in the National Essential Drug List |
| 3 | Unrestricted | Consumption of unrestricted group antibiotics according Measures for the Management of Clinical Application of Antimicrobial Drugs |
| 4 | Restricted | Consumption of restricted group antibiotics according Measures for the Management of Clinical Application of Antimicrobial Drugs |
| 5 | Special | Consumption of special group antibiotics according Measures for the Management of Clinical Application of Antimicrobial Drugs |
| 6 | Urban | Consumption of antibiotic agents in urban hospitals |
| 7 | Primary | Consumption of antibiotic agents in primary hospitals |
| 8 | County | Consumption of antibiotic agents in county hospitals |

**Table S2**. Classification of antibiotics used in this study

| **Antibiotic** | **Class** | **AWaRe category** | **Hierarchical management in China** |
| --- | --- | --- | --- |
| Tetracycline | Tetracyclines | Access | Non-Restricted |
| Doxycycline | Tetracyclines | Access | Non-Restricted |
| Oxytetracycline | Tetracyclines | Watch | Non-Restricted |
| Minocycline | Tetracyclines | Watch/Reserve | Restricted |
| Tigecycline | Glycylcycline | Reserve | Special |
| Chloramphenicol | Chloramphenicols | Access | Restricted |
| Amoxicillin | Broad-spectrum penicillins | Access | Non-Restricted |
| Ampicillin | Broad-spectrum penicillins | Access | Non-Restricted |
| Piperacillin | Broad-spectrum penicillins | Watch | Non-Restricted |
| Azlocillin | Broad-spectrum penicillins | Watch | Restricted |
| Mezlocillin | Broad-spectrum penicillins | Watch | Restricted |
| Sulbenicillin | Broad-spectrum penicillins | Watch | Restricted |
| Ticarcillin | Carbenicillins | Watch | Restricted |
| Penicillin G | Narrow-spectrum penicillins | Access | Non-Restricted |
| Penicillin V | Narrow-spectrum penicillins | Access | Non-Restricted |
| Oxacillin | Narrow-spectrum penicillins | Access | Non-Restricted |
| Cloxacillin | Narrow-spectrum penicillins | Access | Non-Restricted |
| Flucloxacillin | Narrow-spectrum penicillins | Access | Restricted |
| Amoxicillin/Clavulanic  Acid | Broad-spectrum penicillins | Access | Non-Restricted |
| Ampicillin/Sulbactam | Broad-spectrum penicillins | Access | Restricted |
| Piperacillin/Tazobactam | Broad-spectrum penicillins | Watch | Restricted |
| Cefalexin | Cephalosporins | Access | Non-Restricted |
| Cefazolin | Cephalosporins | Access | Non-Restricted |
| Cefradine | Cephalosporins | Access | Non-Restricted |
| Cefadroxil | Cephalosporins | Access | Non-Restricted |
| Cefathiamidine | Cephalosporins | Access | Restricted |
| Cefuroxime | Cephalosporins | Watch | Non-Restricted |
| Cefaclor | Cephalosporins | Watch | Non-Restricted |
| Cefprozil | Cephalosporins | Watch | Restricted |
| Cefotiam | Cephalosporins | Watch | Restricted |
| Ceftriaxone | Cephalosporins | Watch | Non-Restricted |
| Cefotaxime | Cephalosporins | Watch | Restricted |
| Cefixime | Cephalosporins | Watch | Restricted |
| Ceftazidime | Cephalosporins | Watch | Restricted |
| Cefdinir | Cephalosporins | Watch | Restricted |
| Ceftizoxime | Cephalosporins | Watch | Restricted |
| Cefpodoxime Proxetil | Cephalosporins | Watch | Restricted |
| Cefoperazone | Cephalosporins | Watch | Restricted |
| Cefepime | Cephalosporins | Watch | Special |
| Cefpirome | Cephalosporins | Watch | Special |
| Cefmetazole | Cephalosporins | Watch | Restricted |
| Cefoxitin | Cephalosporins | Watch | Restricted |
| Cefminox | Cephalosporins | Watch | Restricted |
| Latamoxef | Cephalosporins | Watch | Restricted |

**Table S2 (Continued)**. Classification of antibiotics used in this study

| Faropenem | Cephalosporins | Reserve | Restricted (Oral)  Special (IV) |
| --- | --- | --- | --- |
| Aztreonam | Monobactams | Reserve | Special |
| Ertapenem | Carbapenems | Watch | Restricted |
| Meropenem | Carbapenems | Watch | Special |
| Imipenem/Cilastatin | Carbapenems | Watch | Special |
| Panipenem/Betamipron | Carbapenems | Watch | Special |
| Biapenem | Carbapenems | Watch | Special |
| Erythromycin | Macrolides | Watch | Non-Restricted |
| Azithromycin | Macrolides | Watch | Non-Restricted  (Oral)  Restricted(IV) |
| Acetylspiramycin | Macrolides | Watch | Non-Restricted |
| Roxithromycin | Macrolides | Watch | Non-Restricted |
| Clarithromycin | Macrolides | Watch | Non-Restricted |
| Dirithromycin | Macrolides | Watch | Restricted |
| Sulfadiazine/Trimethoprim | Trimethoprim | Access | Non-Restricted |
| Trimethoprim | Trimethoprim | Access | Non-Restricted |
| Clindamycin | Macrolide | Access | Non-Restricted |
| Lincomycin | Macrolide | Watch | Non-Restricted |
| Gentamicin | Aminoglycosides | Access | Non-Restricted |
| Amikacin | Aminoglycosides | Access | Non-Restricted |
| Streptomycin | Aminoglycosides | Watch | Non-Restricted |
| Neomycin | Aminoglycosides | Watch | Non-Restricted |
| Tobramycin | Aminoglycosides | Watch | Restricted |
| Etimicin | Aminoglycosides | Watch | Restricted |
| Netilmicin | Aminoglycosides | Watch | Restricted |
| Isepamicin | Aminoglycosides | Watch | Restricted |
| Spectinomycin | Other | Watch | Restricted |
| Ciprofloxacin | Fluoroquinolones | Watch | Non-Restricted |
| Norfloxacin | Fluoroquinolones | Watch | Non-Restricted |
| Levofloxacin | Fluoroquinolones | Watch | Non-Restricted |
| Ofloxacin | Fluoroquinolones | Watch | Non-Restricted |
| Moxifloxacin | Fluoroquinolones | Watch | Restricted |
| Antofloxacin | Fluoroquinolones | Watch | Restricted |
| Lomefloxacin | Fluoroquinolones | Watch | Special |
| Fleroxacin | Fluoroquinolones | Watch | Special |
| Gemifloxacin | Fluoroquinolones | Watch | Special |
| Colistin (oral) | Polymyxins | Reserve | Restricted |
| Colistin (injection) | Polymyxins | Reserve | Special |
| Polymyxin B | Polymyxins | Reserve | Special |
| Metronidazole | Other | Access | Non-Restricted |
| Fosfomycin | Phosphonics | Reserve (IV)/  Watch (Oral) | Non-Restricted |

**Table S2 (Continued)**. Classification of antibiotics used in this study

| Rifampicin | Rifamycins | Watch | Restricted |
| --- | --- | --- | --- |
| Rifaximin | Rifamycins | Watch | Restricted |
| Rifamycin | Rifamycins | Watch | Restricted |
| Fusidic acid | Other | Watch | Special |
| Linezolid | Oxazolidinones | Reserve | Special |
| Daptomycin | Lipopeptides | Reserve | Special |

**Table S3**. Interrupted time series analysis of antibiotic consumption in other categories based on the onset of COVID-19 pandemic as intervention

|  | **Change in level（β_2_）** | | | **Change in level（β_3_）** | | |
| --- | --- | --- | --- | --- | --- | --- |
|  | **Value** | ***p* value** | **95%CI** | **Value** | ***p* value** | **95%CI** |
| Use of relevant antibiotics (expressed in DID) | | | | | | |
| Essential | -2.856 | 0.000 | (-3.935,-1.776) | 0.105 | 0.142 | (-0.037,0.247) |
| Unessential | -1.235 | 0.011 | (-2.167,-0.304) | 0.014 | 0.791 | (-0.090,0.117) |
| Unrestricted | -3.670 | 0.000 | (-5.386,-1.952) | 0.135 | 0.200 | (-0.074,0.345) |
| Restricted | -0.432 | 0.024 | (-0.804,-0.059) | -0.011 | 0.586 | (-0.052,0.030) |
| Special | 0.002 | 0.708 | (-0.007,0.010) | -0.000 | 0.594 | (-0.001,0.001) |
| Urban | -1.249 | 0.008 | (-2.152,-0.347) | -0.028 | 0.660 | (-0.157,0.100) |
| Primary | -2.734 | 0.000 | (-3.956,-1.513) | 0.161 | 0.070 | (-0.014,0.336) |
| County | -0.116 | 0.154 | (-0.277,0.045) | -0.009 | 0.345 | (-0.027,0.010) |

**Table S4**. Interrupted time series analysis of antibiotic expenditures in other categories based on the onset of COVID-19 pandemic as intervention

|  | **Change in level（β_2_’）** | | | **Change in level（β_3_’）** | | |
| --- | --- | --- | --- | --- | --- | --- |
|  | **Value** | ***p* value** | **95%CI** | **Value** | ***p* value** | **95%CI** |
| Use of relevant antibiotics (expressed in DID) | | | | | | |
| Essential | -3.975 | 0.000 | (-6.066,-1.884) | 0.111 | 0.421 | (-0.164,0.385) |
| Unessential | -4.979 | 0.131 | (-11.505,1.546) | -0.054 | 0.878 | (-0.762,0.653) |
| Unrestricted | -4.379 | -2.780 | (-7.552,-1.206) | 0.240 | 1.250 | (-0.149,0.629) |
| Restricted | -4.322 | 0.062 | (-8.879,0.234) | -0.144 | 0.573 | (-0.656,0.367) |
| Special | -0.438 | 0.261 | (-1.214,0.338) | 0.064 | 0.250 | (-0.047,0.176) |
| Urban | -5.853 | 0.023 | (-10.847,-0.859) | 0.127 | 0.654 | (-0.441,0.695) |
| Primary | -1.399 | 0.003 | (-2.311,-0.487) | 0.064 | 0.241 | (-0.044,0.172) |
| County | -1.887 | 0.189 | (-4.740,0.965) | -0.030 | 0.865 | (-0.386,0.325) |

## Supplementary Figures

##
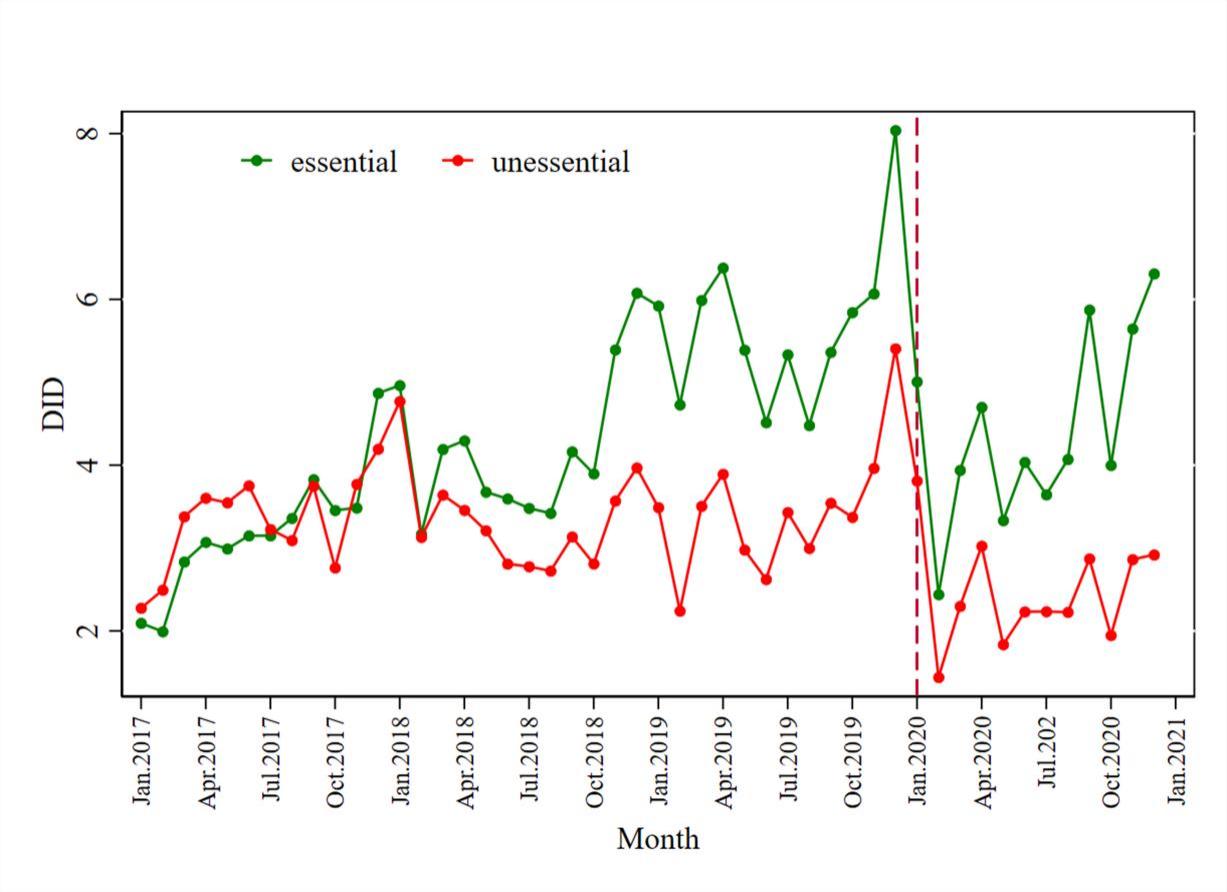


## Supplementary Figure S1. Monthly antibiotic consumption of essential and unessential antibiotics.

##
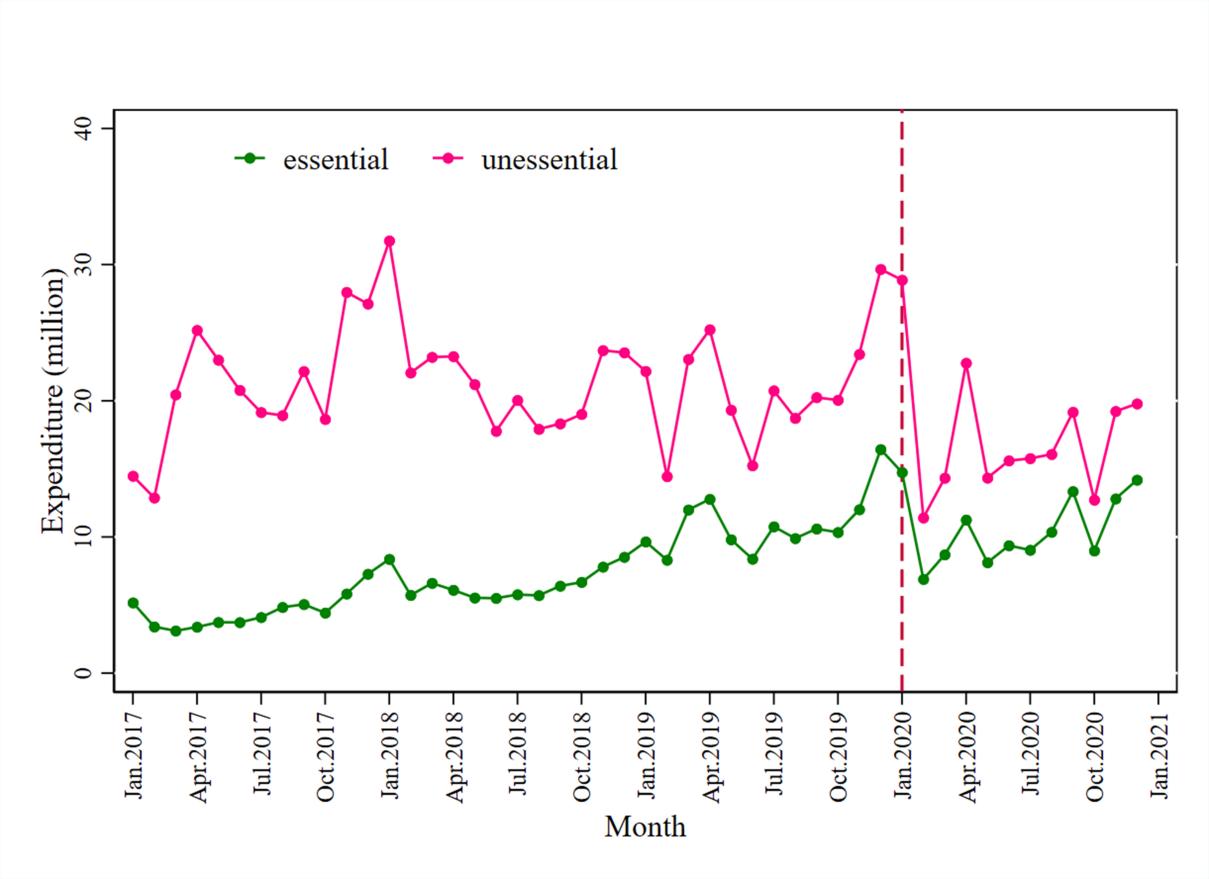


**Supplementary Figure S2.** Monthly antibiotic expenditure of essential and unessential antibiotics.

**
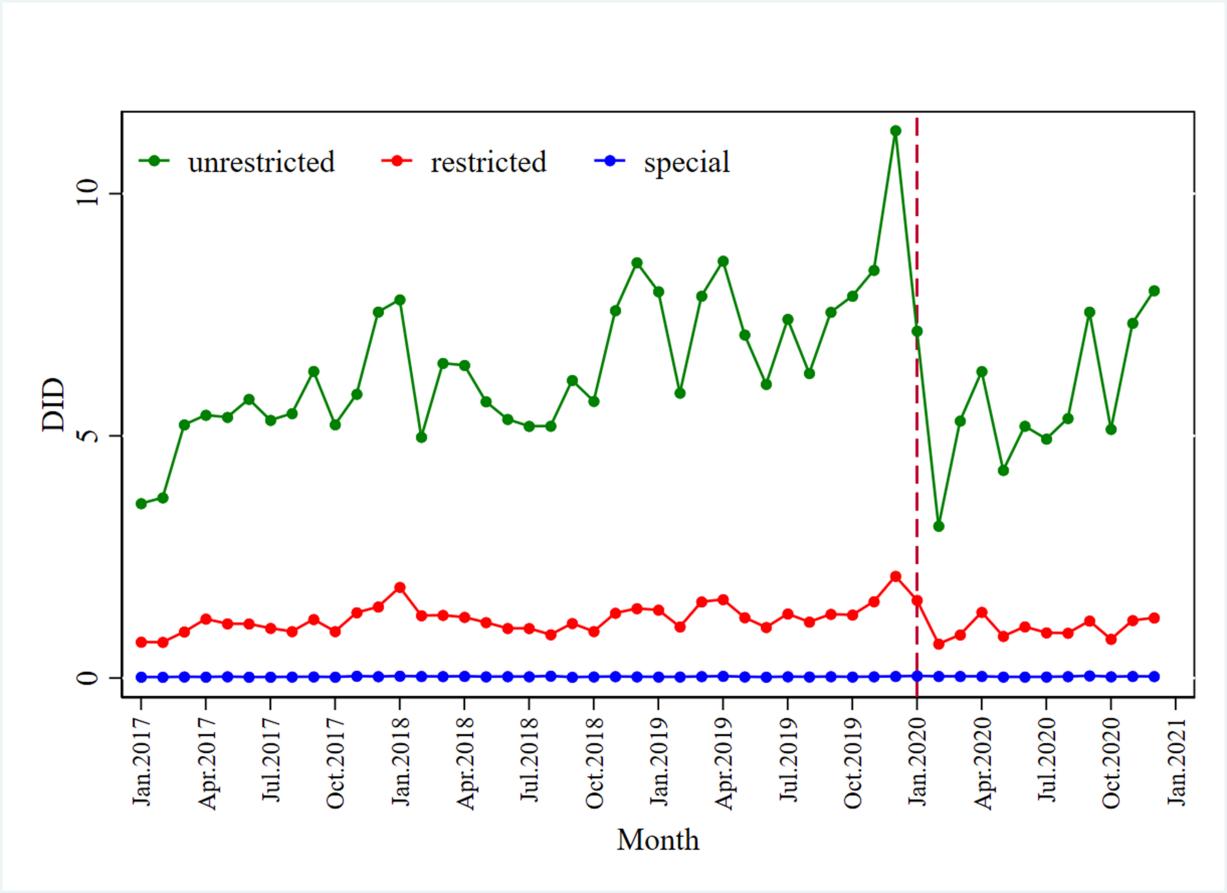
**

**Supplementary Figure S3.** Monthly antibiotic consumption of unrestricted, restricted and special antibiotics.

**
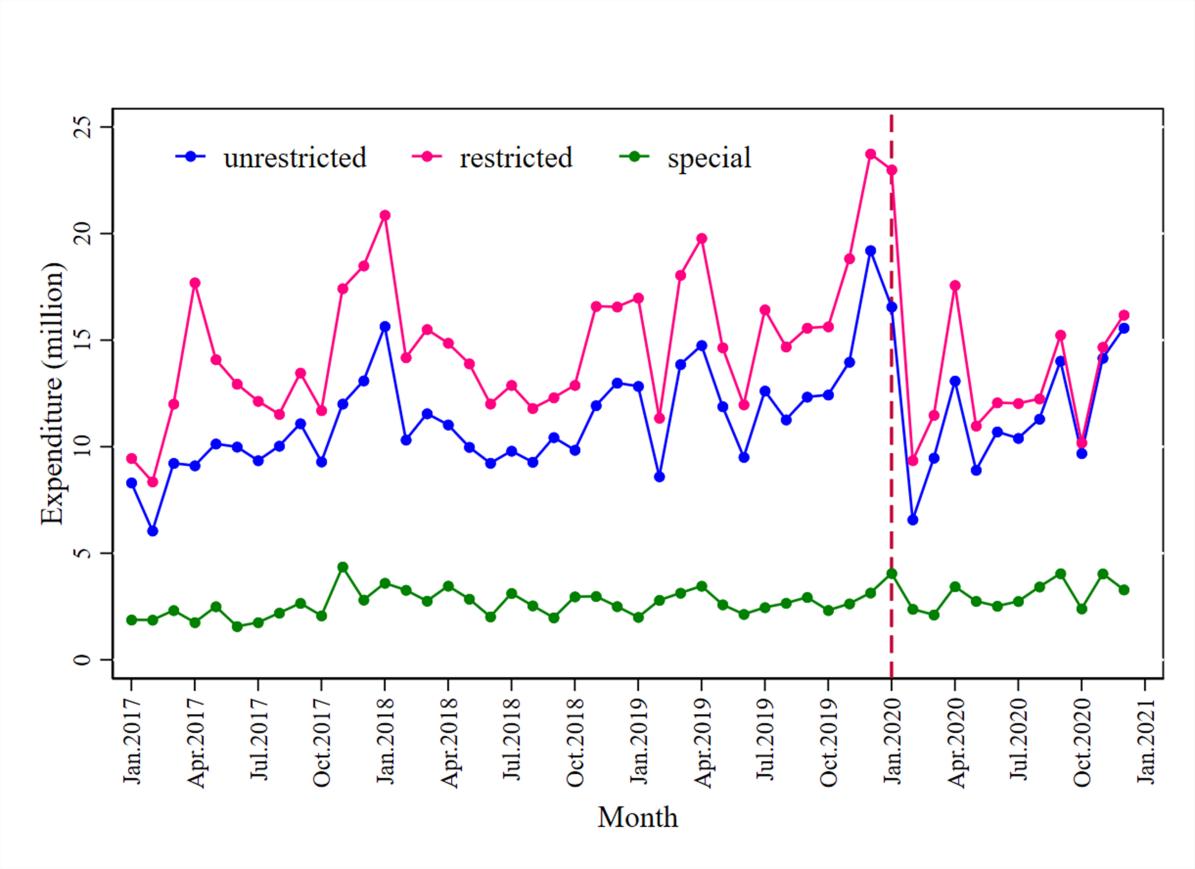
**

**Supplementary Figure S4.** Monthly antibiotic expenditure of unrestricted, restricted and special antibiotics.

**
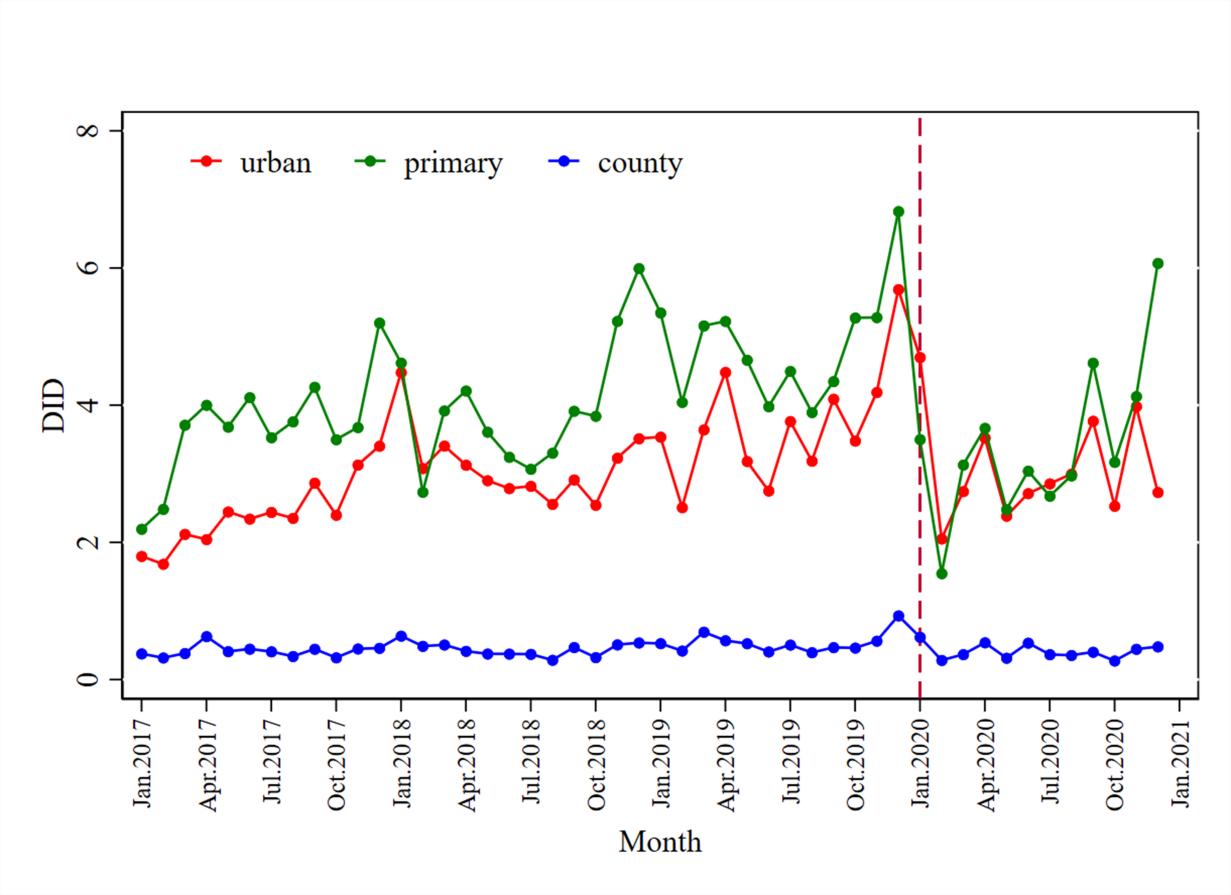
**

**Supplementary Figure S5.** Monthly antibiotic consumption of urban, primary and county antibiotics..

**
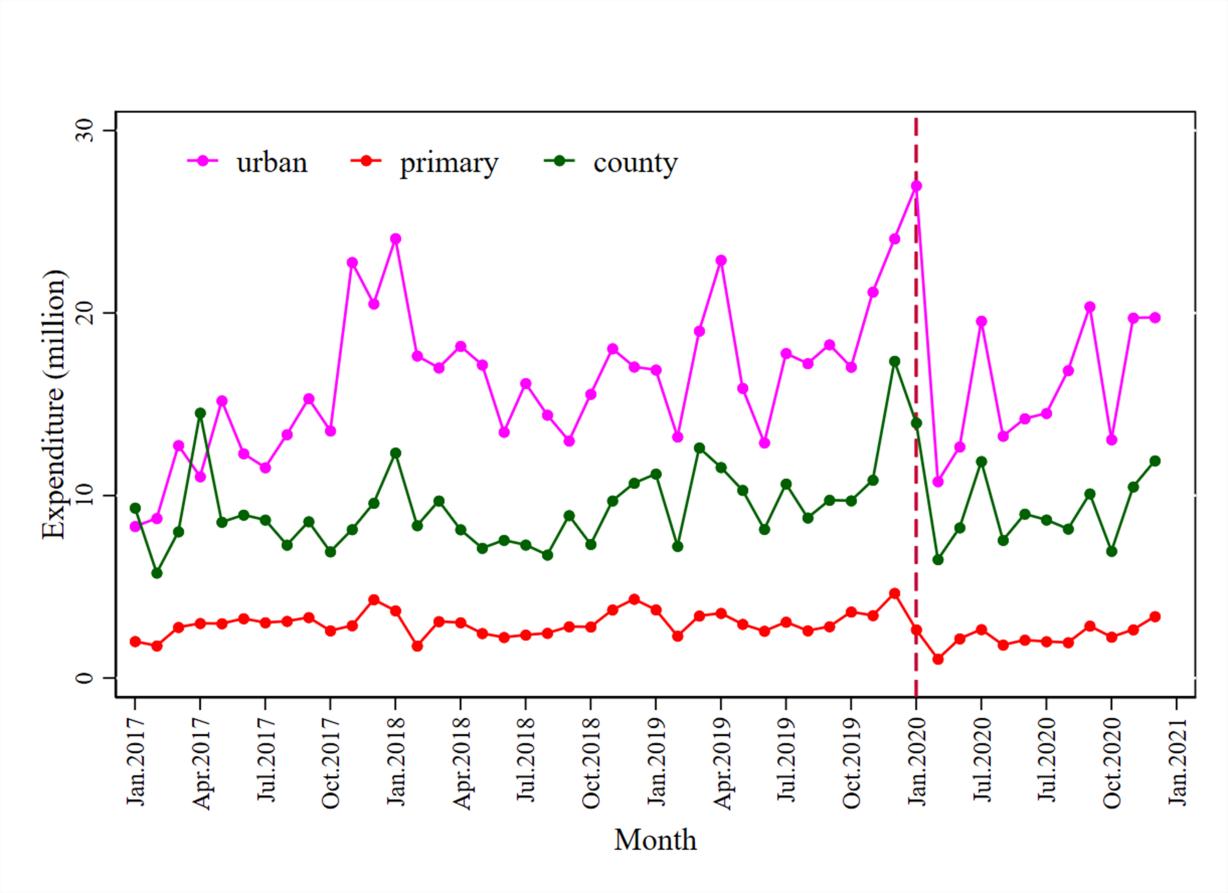
**

**Supplementary Figure S6.** Monthly antibiotic expenditure of urban, primary and county antibiotics.

**
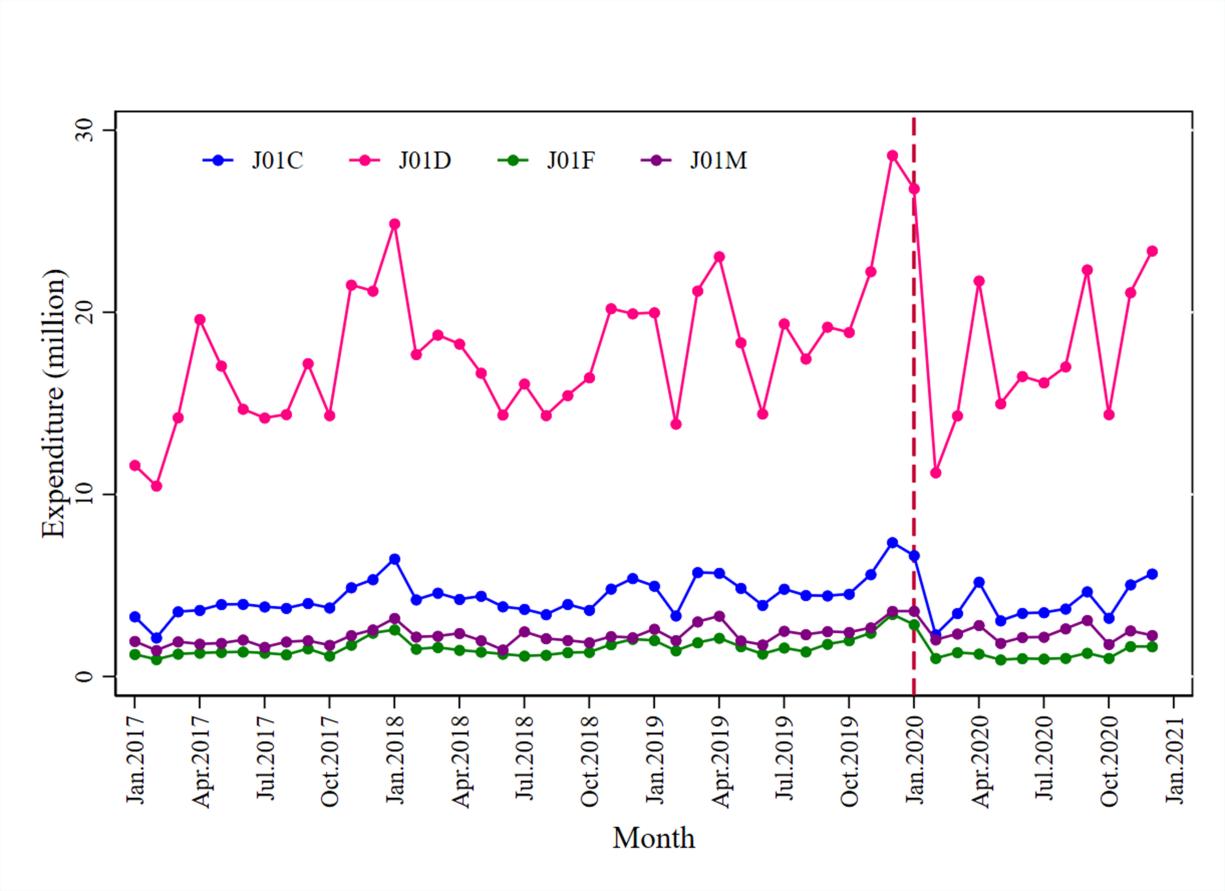
**

**Supplementary Figure S7.** Monthly antibiotic expenditure of penicillins (J01C), cephalosporins (J01D), macrolides, lincosamides, and streptogramins (J01F), and quinolone (J01M).

**
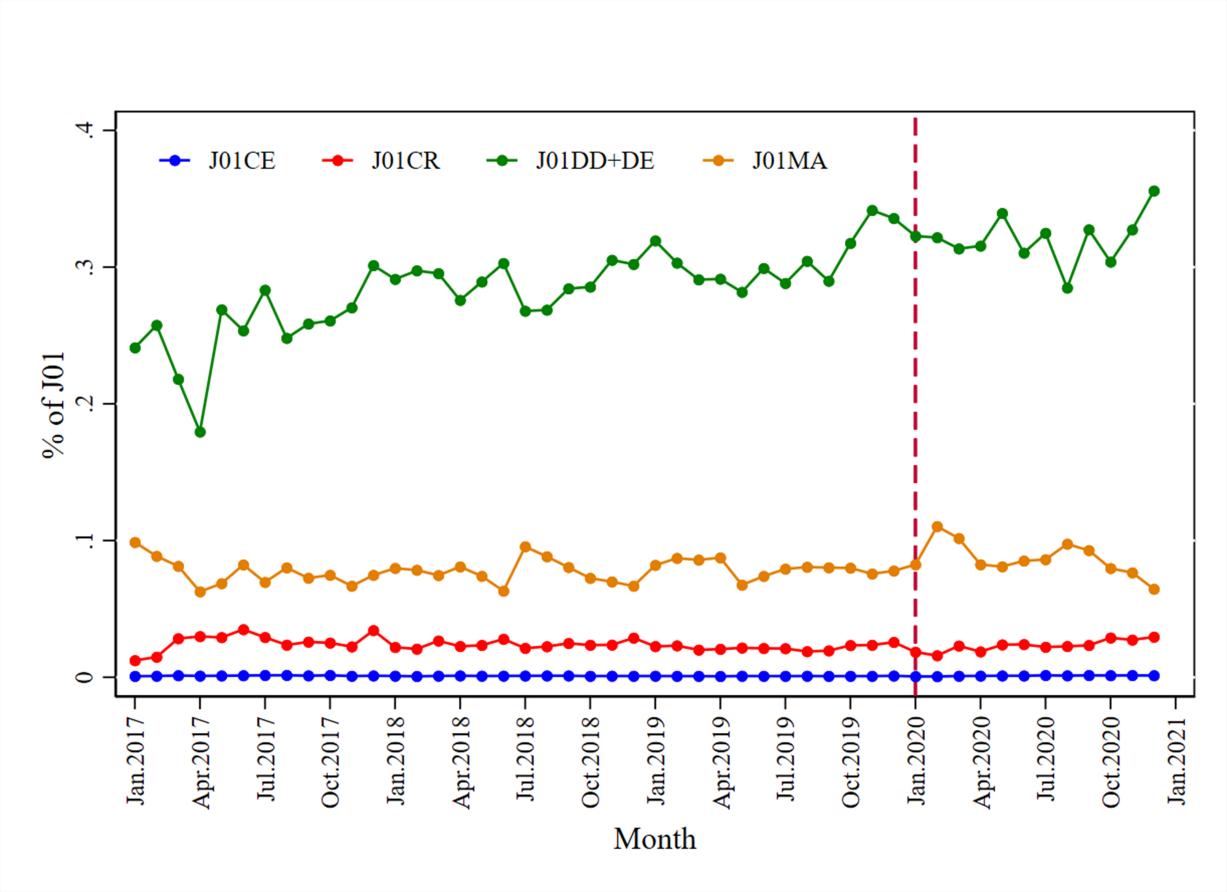
**

**Supplementary Figure S8.** Monthly relative expenditure contributions of J01CE, J01CR, J01(DD+DE), and J01MA to total use of systemic antibiotics.

**
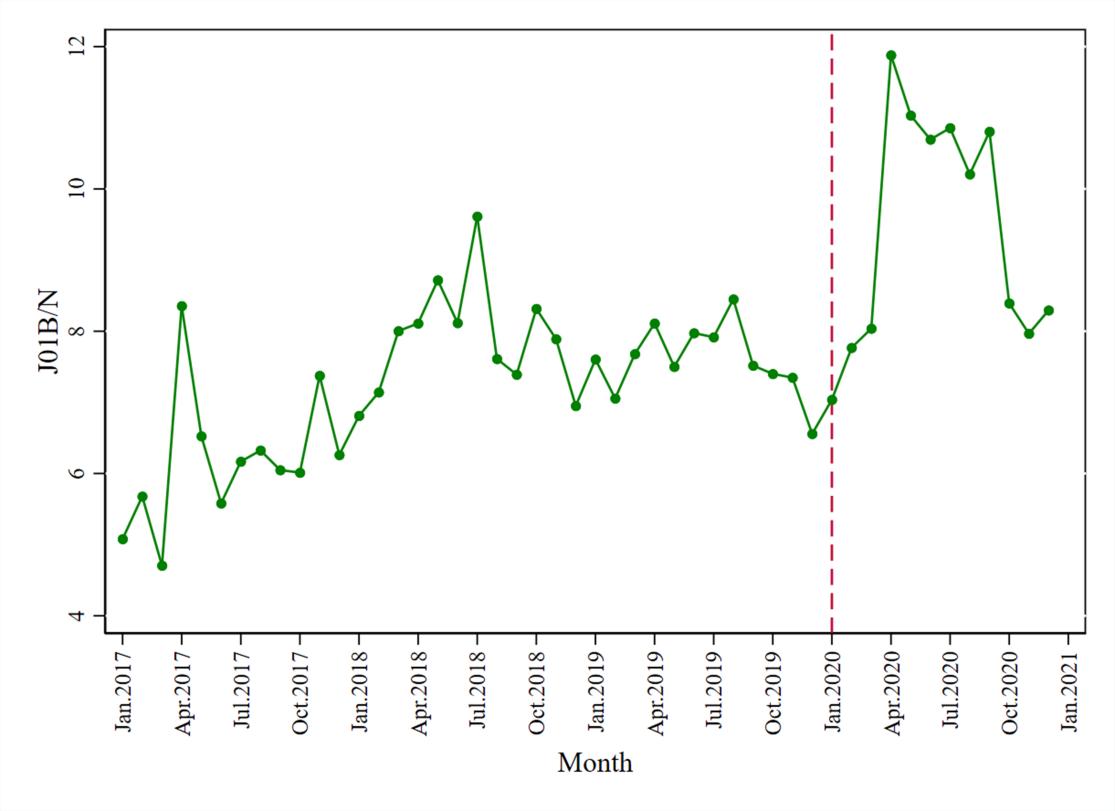
**

**Supplementary Figure S9.** Ratio of expenditure of broad and narrow spectrum antibiotics.
