# Supplementary figures and images for: Determining the impact of the COVID-19 pandemic on the consumption of antibiotics in Shaanxi province, China: an interrupted time-series analysis
Source: Front Public Health. 2025 Feb 19;13:1475207. doi: 10.3389/fpubh.2025.1475207 (PMC11880026; doi:10.3389/fpubh.2025.1475207)

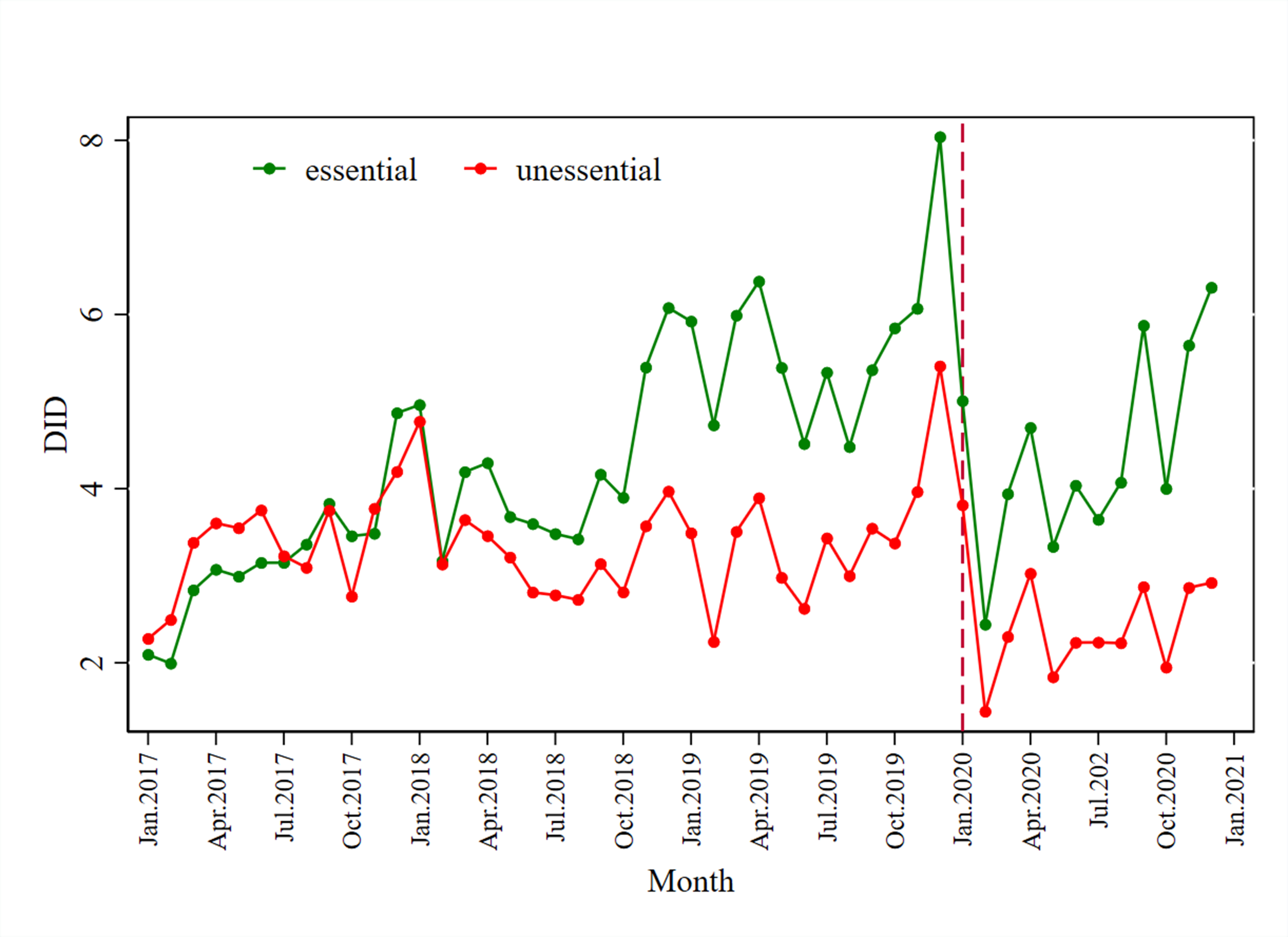

Supplement: Supplementary file 1 [file Data_Sheet_1.zip › Figure S1. Monthly antibiotic consumption of essential and unessential antibiotics.jpg]

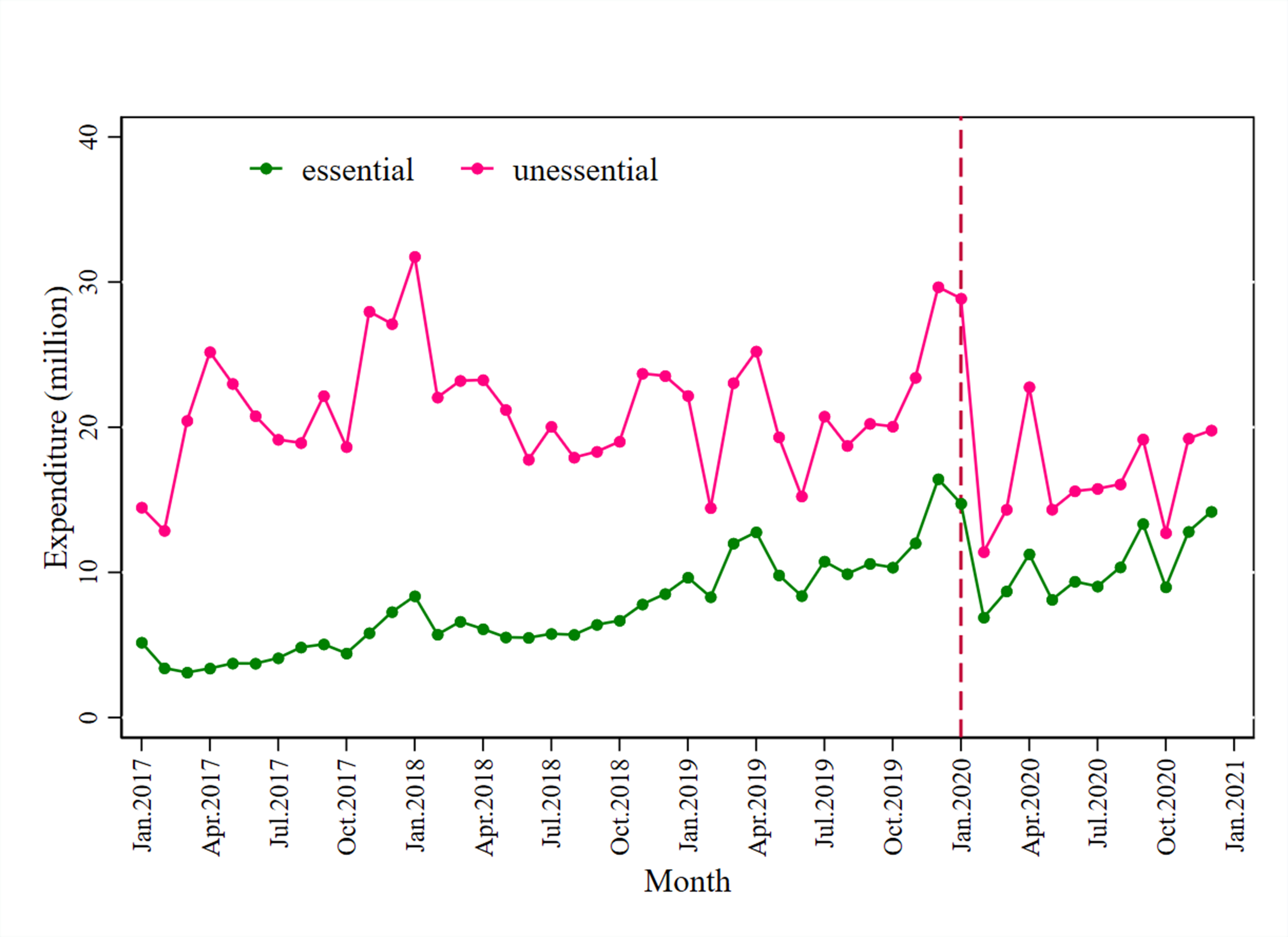

Supplement: Supplementary file 1 [file Data_Sheet_1.zip › Figure S2. Monthly antibiotic expenditure of essential and unessential antibiotics.jpg]

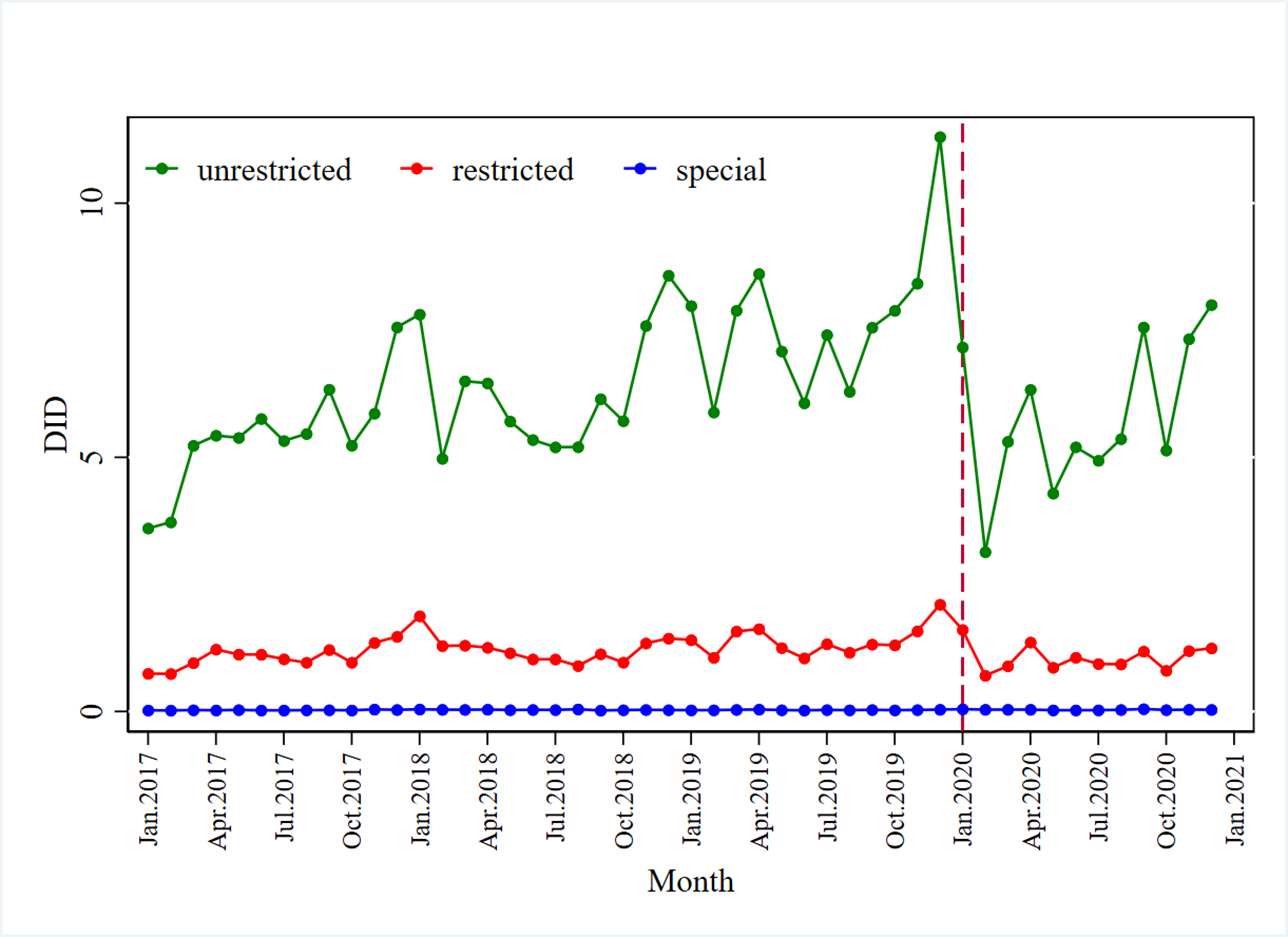

Supplement: Supplementary file 1 [file Data_Sheet_1.zip › Figure S3. Monthly antibiotic consumption of unrestricted, restricted and special antibiotics.jpg]

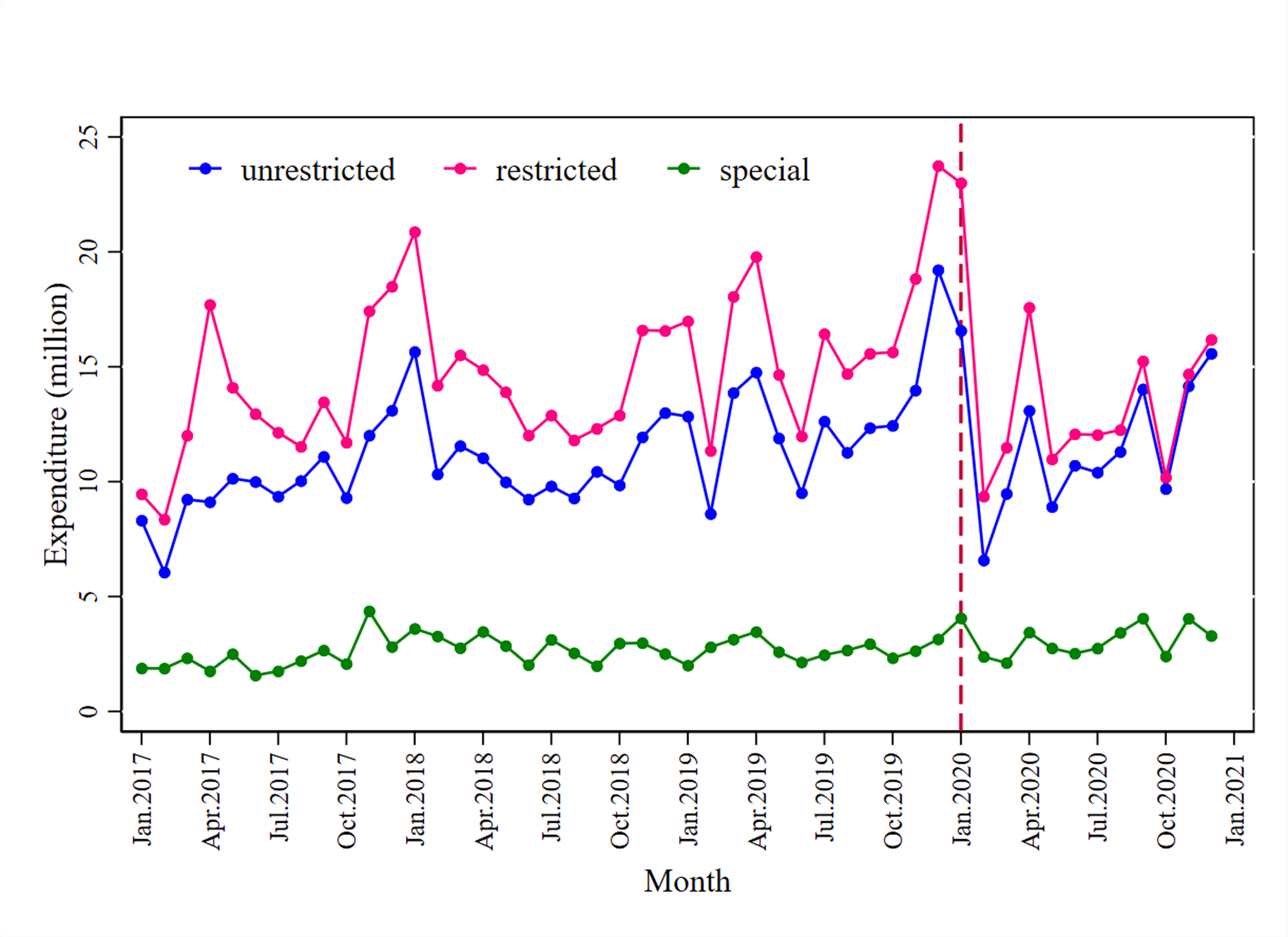

Supplement: Supplementary file 1 [file Data_Sheet_1.zip › Figure S4. Monthly antibiotic expenditure of unrestricted, restricted and special antibiotics.jpg]

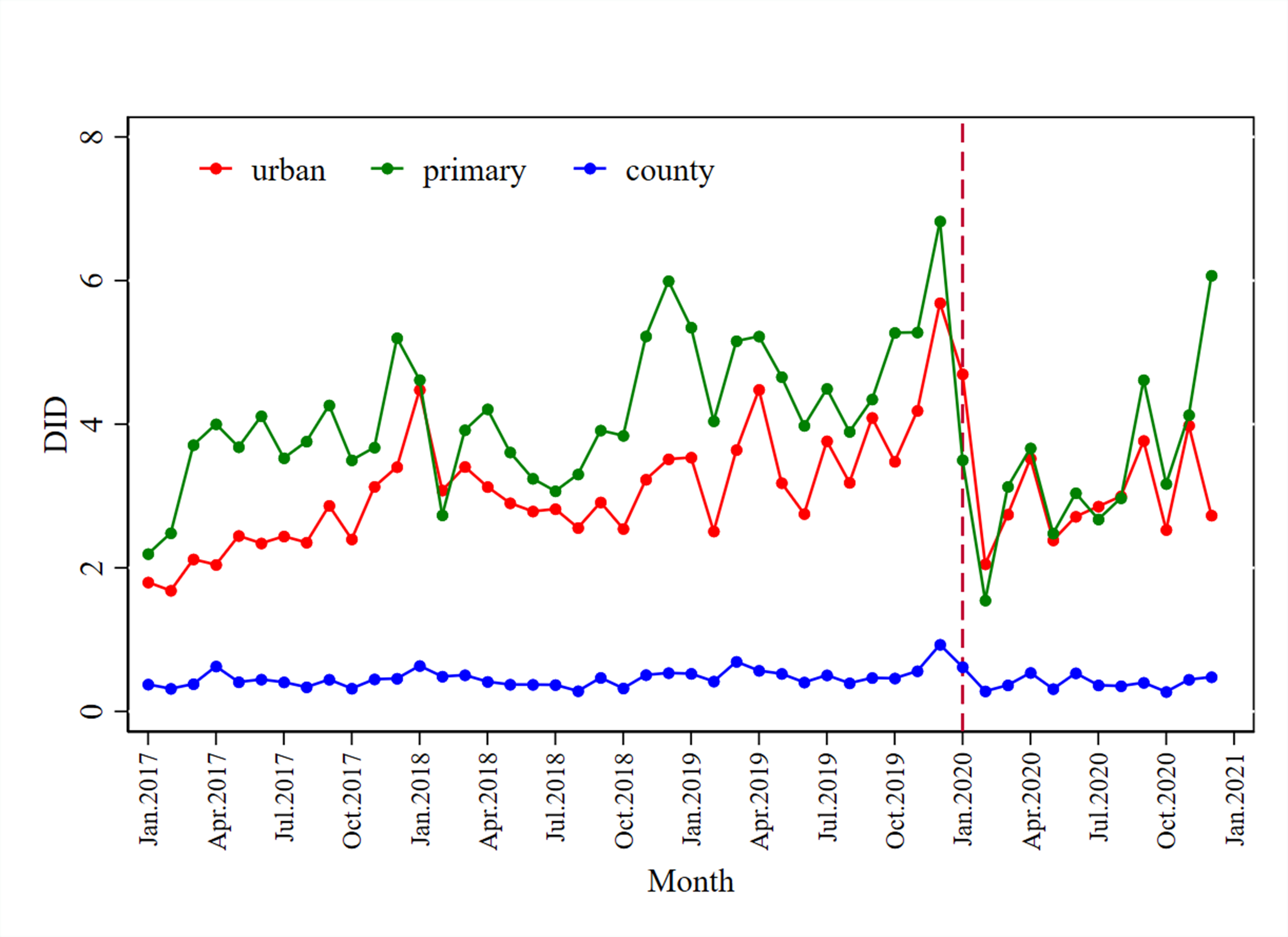

Supplement: Supplementary file 1 [file Data_Sheet_1.zip › Figure S5. Monthly antibiotic consumption of urban, primary and county antibiotics.jpg]

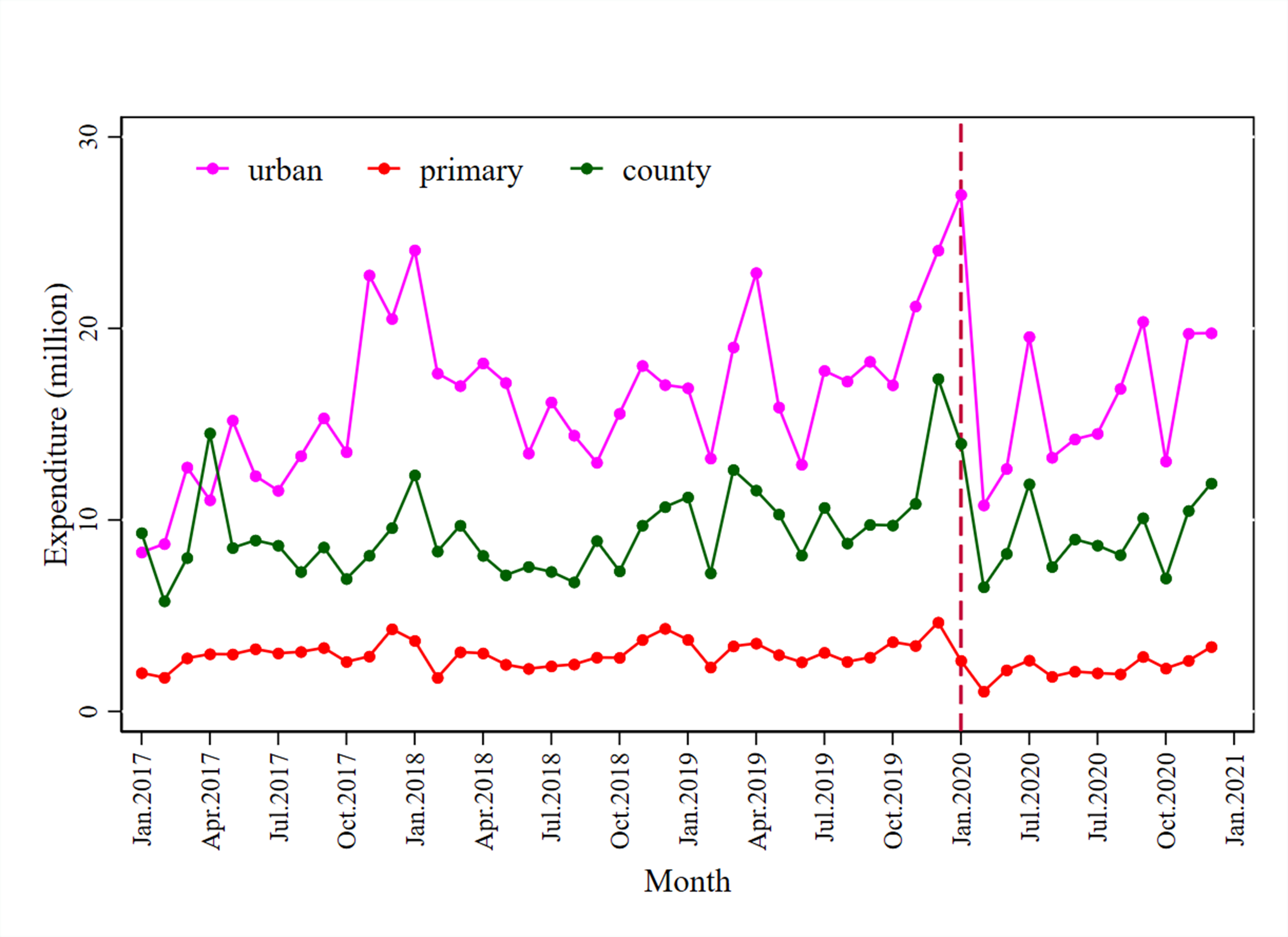

Supplement: Supplementary file 1 [file Data_Sheet_1.zip › Figure S6. Monthly antibiotic expenditure of urban, primary and county antibiotics.jpg]

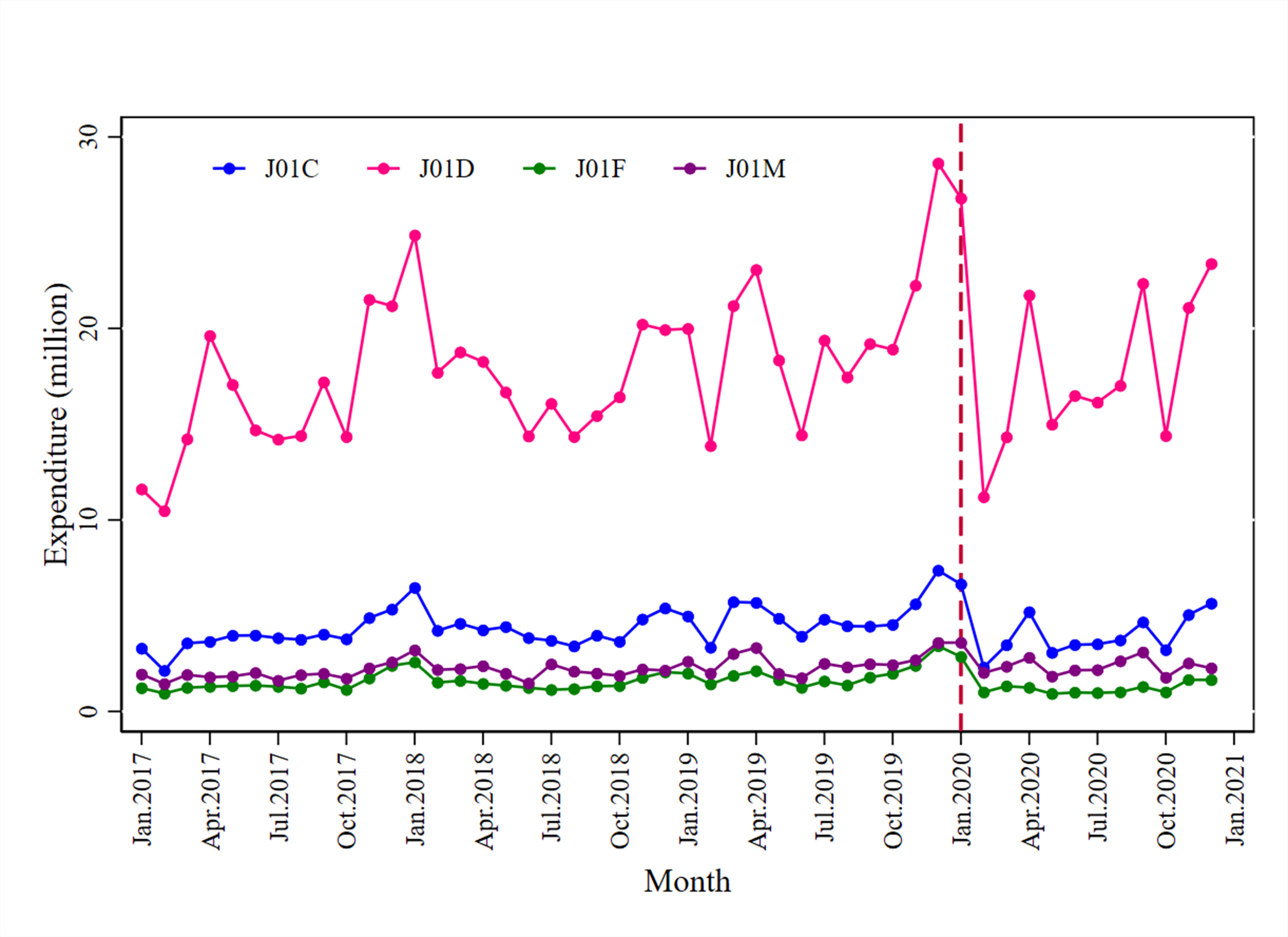

Supplement: Supplementary file 1 [file Data_Sheet_1.zip › Figure S7. Monthly antibiotic expenditure of penicillins (J01C), cephalosporins (J01D), macrolides, lincosamides, and streptogramins (J01F), and quinolone (J01M).jpg]

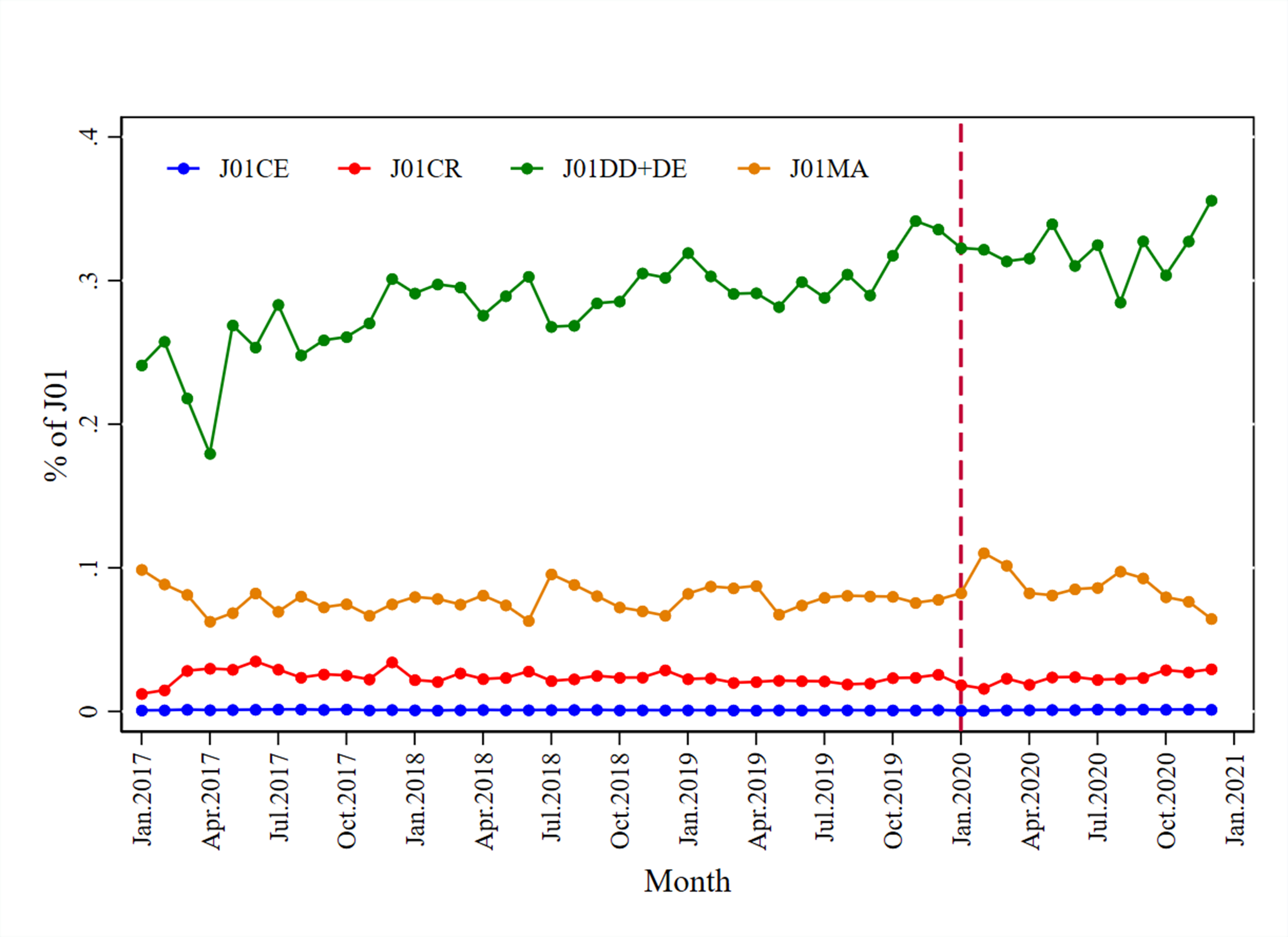

Supplement: Supplementary file 1 [file Data_Sheet_1.zip › Figure S8. Monthly relative expenditure contributions of J01CE, J01CR, J01(DD+DE), and J01MA to total use of systemic antibiotics.jpg]

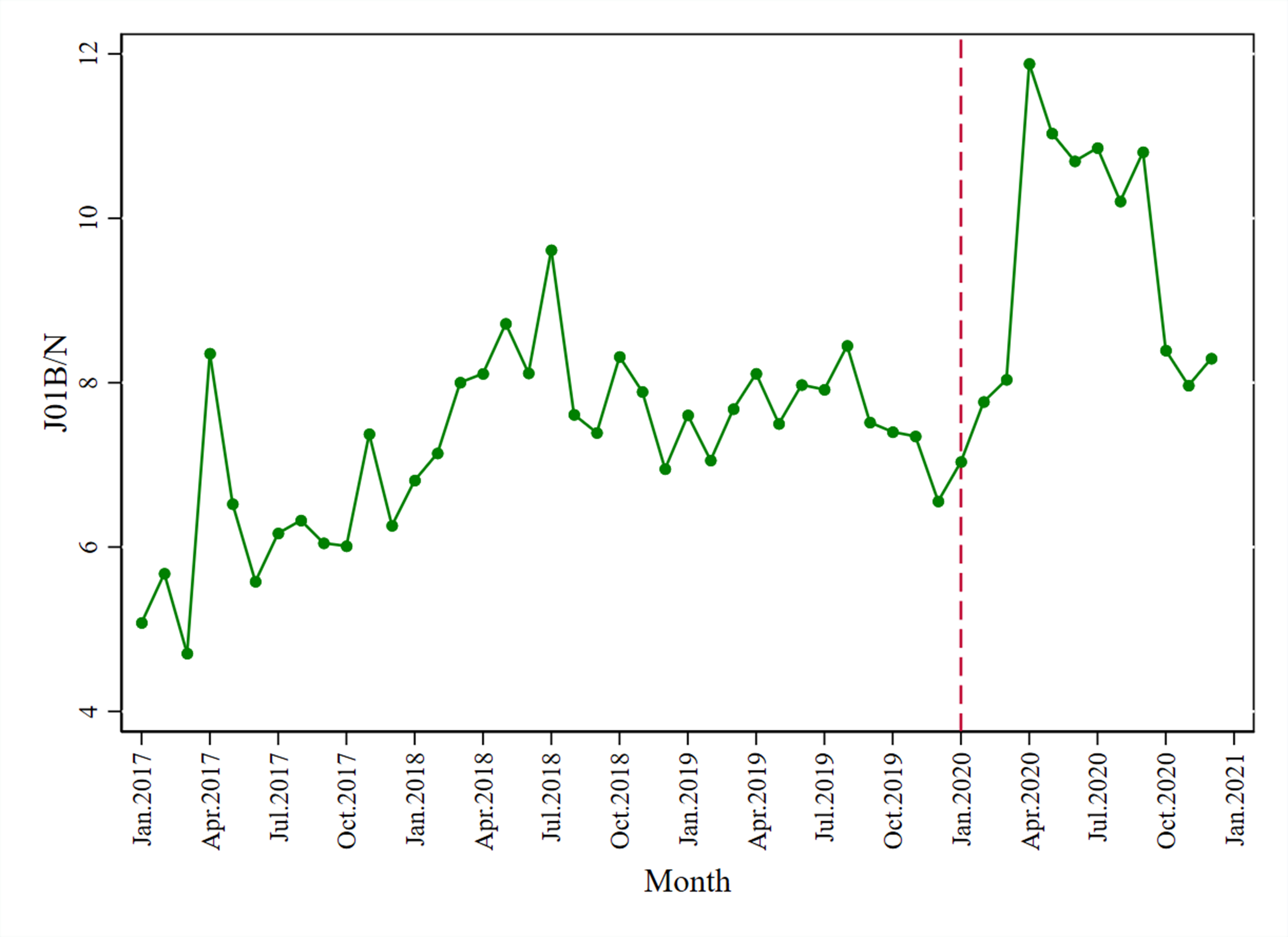

Supplement: Supplementary file 1 [file Data_Sheet_1.zip › Figure S9. Ratio of expenditure of broad and narrow spectrum antibiotics.jpg]
